# Bee-Derived Products in Neurodegenerative Diseases: Bioactive Constituents, Redox-Modulating Mechanisms, Evidence, and Translational Perspectives

**DOI:** 10.3390/molecules31193564

**Published:** 2026-10-07

**Authors:** Jakub Garbarek, Oskar Hys, Alina Woźniak, Karolina Szewczyk-Golec, Jarosław Nuszkiewicz

**Affiliations:** 1 Student Research Club of Medical Biology and Biochemistry, Department of Medical Biology and Biochemistry, Faculty of Medicine, Ludwik Rydygier Collegium Medicum in Bydgoszcz, Nicolaus Copernicus University in Toruń, 24 Karłowicza Street, 85-092 Bydgoszcz, Poland; 312913@stud.umk.pl (J.G.); 335833@stud.umk.pl (O.H.); 2 Department of Medical Biology and Biochemistry, Faculty of Medicine, Ludwik Rydygier Collegium Medicum in Bydgoszcz, Nicolaus Copernicus University in Toruń, 24 Karłowicza Street, 85-092 Bydgoszcz, Poland; karosz@cm.umk.pl

**Keywords:** bee-derived products, oxidative stress, redox signaling, neuroinflammation, neurodegeneration, Nrf2, NF-κB, neuroprotection

## Abstract

Oxidative stress, mitochondrial dysfunction, and chronic neuroinflammation are closely interconnected processes involved in the pathogenesis and progression of neurodegenerative diseases. Bee-derived products, including honey, propolis, royal jelly, bee pollen, bee bread, and bee venom, contain diverse bioactive constituents with antioxidant, anti-inflammatory, immunomodulatory, and redox-modulating properties. This narrative review summarizes the major bioactive compounds present in bee-derived products and evaluates their potential relevance to neuroprotection. Particular attention is given to mechanisms involving activation of the Nrf2/ARE antioxidant response, inhibition of NF-κB-dependent inflammatory signaling, modulation of the NLRP3 inflammasome, mitochondrial protection, regulation of apoptosis, and maintenance of proteostasis. Available evidence from cellular and animal models, together with the limited clinical data, is discussed primarily in the context of Alzheimer’s and Parkinson’s diseases, with additional consideration of amyotrophic lateral sclerosis and other neurodegenerative or neuroinflammatory conditions. Although several bee-derived products and isolated constituents show promising biological effects, their therapeutic translation remains limited by variable chemical composition, insufficient standardization, low or uncertain bioavailability, limited evidence of central nervous system exposure, and safety concerns. Further mechanistic, pharmacokinetic, and well-controlled clinical studies are therefore required before their potential neuroprotective applications can be reliably established.

## 1. Introduction

### 1.1. Oxidative Stress and Neurodegenerative Diseases

Oxidative stress (OS), broadly defined as a disruption of redox homeostasis resulting from an imbalance between the generation of reactive species and the capacity of antioxidant and redox-regulatory systems, is increasingly recognized as an important component of neurodegenerative disease (ND) pathophysiology [1]. Although individual NDs differ in their etiology and clinical manifestations, they share several interconnected pathological features, including mitochondrial dysfunction, neuroinflammation, impaired proteostasis, protein aggregation, and progressive neuronal loss. In this context, OS should not be considered an isolated or universally initiating event, but rather a common contributor to neurodegenerative processes. Importantly, this relationship is bidirectional: oxidative and redox disturbances may promote neurodegeneration, whereas disease-associated mitochondrial dysfunction, inflammation, and proteostatic impairment can further enhance oxidative stress [2].

Reactive oxygen species (ROS) are continuously generated during normal cellular metabolism and also serve important physiological signaling functions. However, excessive ROS generation or insufficient antioxidant defense may shift redox homeostasis toward oxidative damage. In neural tissue, this imbalance can arise from several interconnected processes, particularly mitochondrial dysfunction and activation of neuroinflammatory cells [1]. Sustained oxidative stress may damage lipids, proteins, and nucleic acids, while simultaneously interfering with cellular proteostasis and endogenous antioxidant responses [2].

Persistent redox dysregulation can therefore contribute to a self-amplifying cycle involving mitochondrial impairment, glial activation, neuroinflammation, protein aggregation, and neuronal dysfunction. Such mechanisms are particularly relevant to major neurodegenerative disorders, including Alzheimer’s disease (AD) and Parkinson’s disease (PD), although related processes are also observed in amyotrophic lateral sclerosis (ALS) and other neurodegenerative conditions [2].

### 1.2. Antioxidants, Redox Signaling, and Natural Products

Antioxidant activity should not be understood exclusively as the direct scavenging of free radicals. Although some compounds can directly react with ROS, their concentrations in vivo are often insufficient to provide sustained protection through stoichiometric radical scavenging alone [3]. Increasing attention has therefore been directed toward compounds that influence endogenous antioxidant defenses and redox-sensitive signaling pathways. One of the principal examples is the nuclear factor erythroid 2-related factor 2/antioxidant response element (Nrf2/ARE) pathway. Activation of Nrf2 promotes ARE-dependent transcription of numerous cytoprotective and antioxidant genes, including those encoding heme oxygenase-1 (HO-1), glutathione S-transferases (GSTs), and proteins involved in glutathione homeostasis [4].

Redox regulation is also closely linked to inflammatory signaling. Nuclear factor kappa B (NF-κB) is a redox-sensitive transcription factor with a central role in the expression of pro-inflammatory mediators and in the activation of neuroinflammatory responses. Persistent NF-κB signaling may contribute to ROS and reactive nitrogen species generation and to the maintenance of activated microglial phenotypes, whereas several natural redox-modulating compounds have been reported to attenuate this pathway [5]. In addition to Nrf2 and NF-κB modulation, biologically active compounds may influence mitochondrial function, glial activation, inflammatory mediator production, and cell-survival or cell-death pathways. Persistent disruption of these interconnected systems may amplify oxidative damage, proteostatic stress, inflammation, and neuronal cell death [6].

Natural products constitute an important source of structurally diverse bioactive molecules capable of interacting with such redox-sensitive and inflammatory pathways. Consequently, their biological relevance extends beyond direct antioxidant activity and increasingly encompasses redox pharmacology, chemical biology, and the modulation of endogenous cellular defense mechanisms [7].

### 1.3. Bee-Derived Products

Bee-derived products represent a chemically diverse group of natural biological materials that have attracted considerable interest because of their antioxidant, anti-inflammatory, immunomodulatory, and other biological activities [8,9,10]. Their potential relevance to disorders associated with redox dysregulation derives from the presence of multiple classes of bioactive constituents, including phenolic acids, flavonoids, caffeic acid phenethyl ester (CAPE), peptides, proteins, lipids, organic acids, enzymatic components, and other low-molecular-weight compounds [11,12].

The principal bee-derived products include honey, propolis, royal jelly, bee pollen, bee bread, bee venom, and beeswax, together with less extensively studied materials of bee origin [13,14]. Their chemical composition and biological properties vary substantially among products and are further influenced by botanical and geographical origin, processing, and storage. Nevertheless, several bee-derived products have demonstrated antioxidant or redox-related activity in chemical, cellular, and experimental models [11,15,16].

Importantly, their potential biological effects should not be reduced to direct ROS scavenging. Depending on the product and its constituents, bee-derived materials may influence oxidative, inflammatory, and immune processes simultaneously, thereby affecting multiple components of cellular homeostasis [12,17]. This multi-target profile provides the rationale for investigating bee-derived products and their constituent compounds in conditions characterized by chronic redox dysregulation and neuroinflammation.

### 1.4. Aim and Scope of the Review

Interest in the biological properties of bee-derived products has increased considerably, and antioxidant activity has been demonstrated for selected products and preparations in chemical and cellular models [18]. However, translation of these observations into biologically relevant and clinically applicable neuroprotective strategies remains challenging. Major limitations include the substantial chemical heterogeneity of natural products, insufficient standardization of preparations, incomplete characterization of molecular mechanisms, variable bioavailability and metabolism, and uncertainty regarding whether bioactive constituents or their metabolites reach biologically relevant concentrations within the central nervous system.

Bee-derived products such as honey, propolis, royal jelly, bee pollen, bee bread, bee venom, and beeswax contain chemically diverse constituents with potentially relevant biological activities [19,20,21,22]. Their interactions with oxidative stress, inflammatory signaling, mitochondrial function, and neuronal survival make them of particular interest in the context of neurodegenerative disorders, especially AD and PD, while emerging evidence also concerns ALS and other neurodegenerative or neuroinflammatory conditions [23].

Accordingly, this narrative and mechanistic review aims to summarize the major bee-derived products and their bioactive constituents; critically evaluate their antioxidant and redox-modulating properties; examine molecular mechanisms potentially relevant to neuroprotection, including modulation of Nrf2/ARE- and NF-κB-dependent signaling, mitochondrial function, neuroinflammation, proteostasis, and cell-survival pathways; and assess the available evidence from cellular, animal, and human studies. Although particular attention is given to redox-related mechanisms, the review is not restricted to effects that can be directly attributed to oxidative stress modulation. Other experimentally supported neurobiologically relevant mechanisms, as well as beneficial effects for which the underlying molecular pathways remain incompletely defined, are also considered where appropriate. Particular attention is given to the distinction between chemical antioxidant activity and biologically relevant redox modulation, as well as to pharmacokinetic limitations, blood–brain barrier relevance, standardization, safety, and other challenges affecting translation. Finally, current gaps in the evidence are identified to define priorities for future mechanistic, pharmacokinetic, and clinical research.

Relevant literature for this narrative review was identified through targeted searches of PubMed, Web of Science Core Collection, and Google Scholar. Search terms combined the names of major bee-derived products and representative constituents with terms related to neurodegenerative diseases, neuroprotection, oxidative stress and redox signaling, neuroinflammation, mitochondrial dysfunction, apoptosis, proteostasis, and relevant molecular pathways. No predefined lower publication-date limit was applied, and the literature search was updated through October 2026. Publications were prioritized according to their direct relevance to the scope of the review, methodological appropriateness, recency, and contribution to mechanistic, preclinical, or clinical interpretation. Where available, original cellular, animal, and human studies were prioritized for the evaluation of specific biological effects, while review articles were used to provide broader mechanistic and contextual background; computational studies were included when they provided relevant hypothesis-generating evidence. Publications outside the scope of the review or not available in English were excluded. As this was a narrative rather than a systematic review, no formal PRISMA-based screening procedure or quantitative risk-of-bias assessment was applied.

## 2. Redox and Neuroinflammatory Framework for Evaluating Bee-Derived Neuroprotection

This section provides a concise mechanistic framework for interpreting the redox- and inflammation-related effects of bee-derived products discussed in subsequent sections. It is not intended as a comprehensive review of oxidative stress and neuroinflammation in neurodegenerative diseases, but focuses on the pathways and processes directly relevant to the mechanistic evaluation of bee-derived products and their constituents.

### 2.1. Major Sources of ROS in the Nervous System

Mitochondrial metabolism represents a major source of reactive oxygen species (ROS) in neural tissue, and mitochondrial dysfunction can markedly increase ROS generation [24]. Additional enzymatic sources include NADPH oxidases (NOX), which generate ROS as part of their physiological and pathological signaling functions [25,26,27]. Excitotoxic stimulation and excessive intracellular Ca^2+^ loading can further impair mitochondrial function, promote electron leakage, and increase ROS production, thereby linking neuronal hyperactivity with redox dysregulation [28,29,30].

Redox-active metal dyshomeostasis provides another mechanism of oxidative injury. Iron and copper can catalyze Fenton-type reactions and promote formation of highly reactive oxygen species, while disturbances in other metal-dependent processes may indirectly disrupt cellular redox homeostasis [31,32]. These mechanisms can converge with mitochondrial dysfunction and inflammatory signaling to amplify oxidative stress.

Impaired proteostasis is closely interconnected with redox dysregulation. Oxidative modifications can interfere with protein folding and degradation, whereas accumulation of misfolded or aggregated proteins can further impair mitochondrial function and enhance ROS generation [33]. This bidirectional relationship is relevant to major neurodegenerative diseases, including amyloid-β and tau pathology in Alzheimer’s disease, α-synuclein accumulation in Parkinson’s disease, and mutant superoxide dismutase 1 (SOD1)-associated pathology in amyotrophic lateral sclerosis.

### 2.2. Endogenous Antioxidant Defense Systems

Endogenous antioxidant and redox-regulatory systems maintain cellular redox homeostasis while preserving physiological ROS signaling. These defenses include enzymatic components such as superoxide dismutase (SOD), catalase (CAT), glutathione peroxidase (GPx), glutathione reductase, peroxiredoxins, and thioredoxin reductases, together with non-enzymatic systems including glutathione and vitamins C and E [34,35,36,37,38,39,40]. Collectively, these systems control superoxide, hydrogen peroxide, organic hydroperoxides, and protein thiol redox states across different cellular compartments.

An important inducible component of this defense network is the Nrf2/ARE pathway. Under basal conditions, Kelch-like ECH-associated protein 1 (Keap1) promotes Nrf2 degradation, whereas oxidative or electrophilic modification of Keap1 stabilizes Nrf2 and enables its nuclear accumulation and binding to antioxidant response elements [41,42]. This promotes transcription of cytoprotective genes including HO-1, NAD(P)H quinone dehydrogenase 1 (NQO1), GSTs, and proteins involved in glutathione and thiol-redox homeostasis. Because several bee-derived constituents have been reported to modulate Nrf2-associated signaling, this pathway is considered in greater detail in Section 5.

### 2.3. Redox Signaling Versus Oxidative Damage

ROS are not exclusively harmful metabolic by-products but also function as physiological signaling molecules. Under controlled conditions, they participate in reversible redox modifications that regulate cellular processes, while endogenous redox-regulatory systems restrict their concentration, localization, and duration of action [43]. Hydrogen peroxide is particularly important in redox signaling because it can reversibly oxidize cysteine residues in redox-sensitive proteins and thereby modify the activity of enzymes, transcription factors, and signaling proteins [44]. Other reactive species may likewise participate in redox-sensitive cellular responses [45].

The biological consequences of ROS therefore depend on their chemical identity, concentration, localization, and duration of exposure. Controlled and reversible ROS signaling is commonly described as oxidative eustress, whereas excessive or sustained ROS production that overwhelms redox-regulatory capacity and causes macromolecular damage constitutes oxidative distress [46]. Accordingly, oxidative modifications should not automatically be equated with cellular injury, as spatially and temporally controlled redox changes are integral components of physiological signaling [46].

### 2.4. Neuroinflammation and Redox-Dependent Glial Activation

Neuroinflammation is closely coupled to redox dysregulation through NF-κB-dependent signaling, inflammasome activation, and glial responses. In the canonical NF-κB pathway, IKK-dependent degradation of IκB permits NF-κB nuclear translocation and transcription of pro-inflammatory mediators, including iNOS, COX-2, TNF-α, IL-1β, and IL-6 [47].

NF-κB-dependent priming also facilitates activation of the NLR family pyrin domain containing 3 (NLRP3) inflammasome, which can be triggered by mitochondrial ROS and other cellular danger signals. NLRP3 assembly activates caspase-1 and promotes maturation of IL-1β and IL-18, thereby linking mitochondrial dysfunction and oxidative stress with inflammatory signaling [48]. In turn, inflammatory pathways can further increase ROS and reactive nitrogen species generation, whereas Nrf2-dependent responses provide an important counter-regulatory antioxidant and anti-inflammatory mechanism [49].

Persistent redox-inflammatory signaling can promote reactive states in microglia and astrocytes, increasing the production of inflammatory mediators and reactive species, while signals released from damaged neurons can further sustain glial activation [50,51]. Neuroinflammation and neuronal injury should therefore be regarded as mutually reinforcing processes. This redox-inflammatory network provides the mechanistic framework for evaluating the effects of bee-derived products and constituents discussed in subsequent sections.

**Figure 1:**
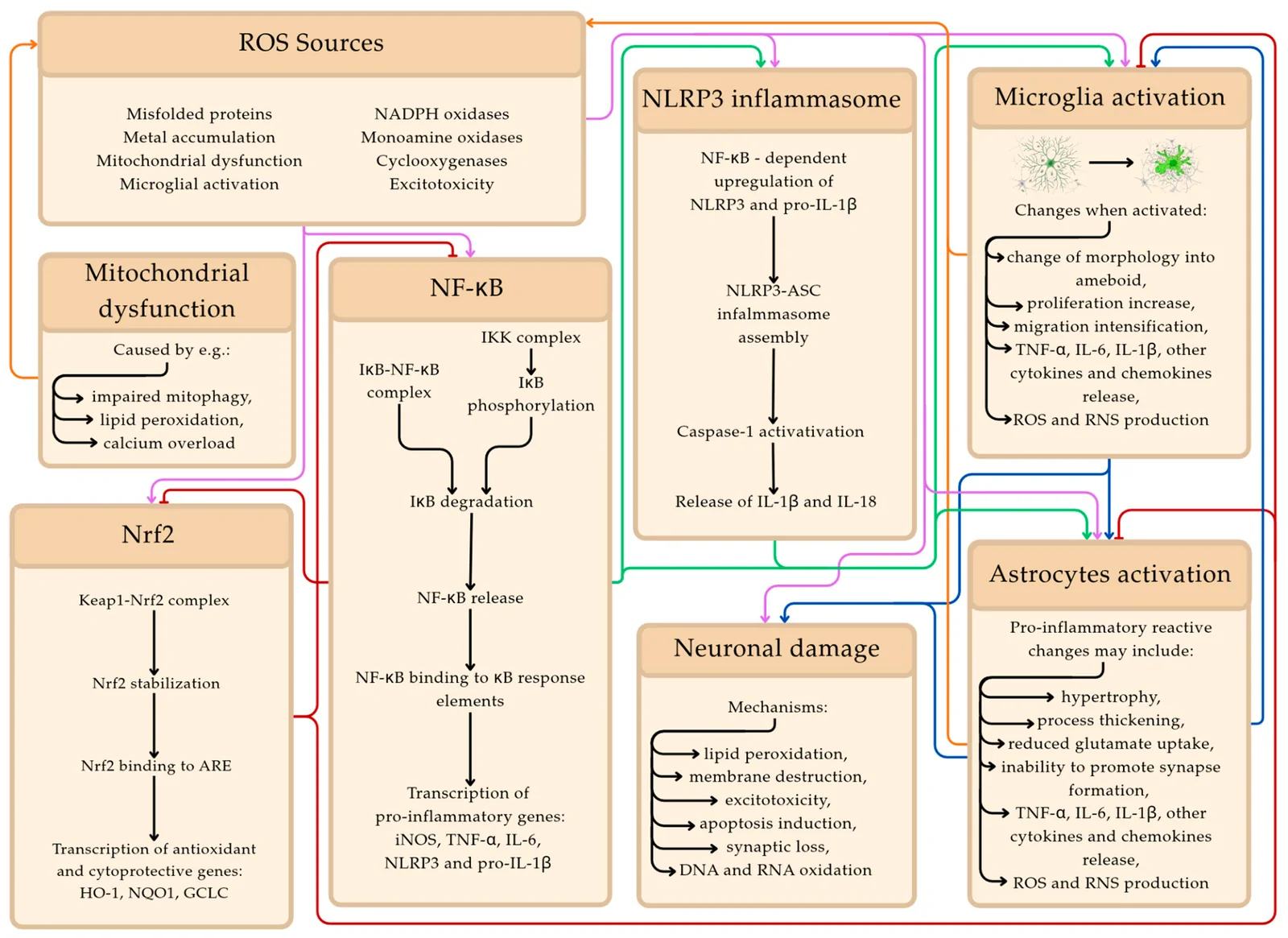
Redox dysregulation and neuroinflammatory signaling in neurodegenerative diseases. Major sources and drivers of reactive oxygen species (ROS), including mitochondrial dysfunction, redox-active metal dyshomeostasis, enzymatic ROS generation, excitotoxicity, and glial activation, contribute to oxidative injury and neuronal damage. Oxidative and inflammatory signals interact with nuclear factor erythroid 2-related factor 2 (Nrf2), nuclear factor κB (NF-κB), and NLR family pyrin domain containing 3 (NLRP3) inflammasome signaling, while activated microglia and reactive astrocytes can further amplify cytokine production and ROS/reactive nitrogen species (RNS) generation. Nrf2-dependent transcription represents an important counter-regulatory antioxidant and cytoprotective response. The scheme provides a simplified representation of the major interconnected redox and inflammatory processes relevant to neurodegeneration. Arrowheads indicate the direction of the depicted processes or interactions. Black arrows illustrate simplified relationships within individual panels, whereas colored connecting arrows link interacting processes across panels; the colors are used only to facilitate visual tracking and do not denote distinct classes of molecular regulation. Abbreviations: ARE, antioxidant response element; ASC, apoptosis-associated speck-like protein containing a caspase activation and recruitment domain; DNA, deoxyribonucleic acid; GCLC, glutamate-cysteine ligase catalytic subunit; HO-1, heme oxygenase-1; IκB, inhibitor of nuclear factor κB; IKK, IκB kinase; IL, interleukin; iNOS, inducible nitric oxide synthase; Keap1, Kelch-like ECH-associated protein 1; NADPH, reduced nicotinamide adenine dinucleotide phosphate; NF-κB, nuclear factor κB; NLRP3, NLR family pyrin domain containing 3; NQO1, NAD(P)H quinone dehydrogenase 1; Nrf2, nuclear factor erythroid 2-related factor 2; RNA, ribonucleic acid; RNS, reactive nitrogen species; ROS, reactive oxygen species; TNF-α, tumor necrosis factor-α.

The interconnected relationships among ROS generation, mitochondrial dysfunction, Nrf2- and NF-κB-dependent signaling, NLRP3 inflammasome activation, glial reactivity, and neuronal injury are summarized in Figure 1.

## 3. Bee-Derived Products as Sources of Antioxidant and Redox-Modulating Compounds

Bee-derived products are chemically heterogeneous biological matrices whose composition and redox-related properties vary substantially according to product type, botanical and geographical origin, processing, extraction conditions, and analytical methodology [52,53,54,55,56,57]. This variability is particularly pronounced in honey, propolis, bee pollen, and bee bread, in which differences in floral source and processing can markedly affect the abundance and profile of phenolic compounds and other bioactive constituents. Consequently, individual bee-derived products should not be regarded as chemically uniform materials, and comparisons between studies require consideration of product origin, chemical characterization, sample preparation, and analytical methodology.

Accordingly, the following subsections characterize the major bee-derived products with emphasis on their principal bioactive constituents, general antioxidant and redox-related properties, evidence base, and product-specific limitations. Detailed molecular mechanisms relevant to neuroprotection are considered separately in subsequent sections.

### 3.1. Honey

Honey is a complex natural matrix composed predominantly of carbohydrates and water, but it also contains numerous minor constituents, including organic acids, minerals, amino acids, enzymes, volatile compounds, and phenolic compounds [57,58]. The qualitative and quantitative composition of these minor components varies substantially depending on botanical and geographical origin, environmental conditions, and processing. Among the compounds most relevant to antioxidant and redox-related properties are phenolic acids, including caffeic, *p*-coumaric, and ferulic acids, as well as flavonoids such as pinocembrin, kaempferol, chrysin, rutin, quercetin, and luteolin [58,59,60,61].

The antioxidant capacity of honey has been extensively characterized using chemical and in vitro approaches. Total phenolic content (TPC) and total flavonoid content (TFC) frequently correlate with antioxidant capacity assessed using assays such as 2,2-diphenyl-1-picrylhydrazyl (DPPH), 2,2′-azino-bis(3-ethylbenzothiazoline-6-sulfonic acid) (ABTS), and ferric reducing antioxidant power (FRAP) [57,60,62,63]. Phenolic compounds are considered major contributors to these effects, although organic acids, enzymes, minerals, and other minor constituents may also contribute to the overall antioxidant properties of honey [57,64]. However, TPC values determined using the Folin–Ciocalteu assay should be interpreted cautiously because the reagent is not specific to phenolic compounds. In honey, high concentrations of reducing sugars and other non-phenolic reducing constituents may contribute to the measured response and lead to overestimation of the true phenolic content [65].

Botanical origin is a major determinant of the phenolic profile and measured antioxidant capacity of honey. Darker and honeydew honeys often show higher phenolic content and antioxidant activity than lighter blossom honeys, although this relationship is not universal [59,62,66]. Honey color alone should therefore not be regarded as a reliable surrogate for biological activity. Representative compositional and antioxidant characteristics of major honey categories are summarized in Table 1.

Importantly, chemical antioxidant activity should not be equated directly with biological or neuroprotective efficacy. The biological relevance of honey depends on the composition and characterization of the investigated preparation and on whether its constituents achieve biologically meaningful effects in cellular or in vivo systems [61,63].

### 3.2. Propolis

Propolis is a resinous bee-derived material produced from plant resins and exudates collected by bees and mixed with wax and bee secretions. Its chemical composition is highly complex and varies considerably depending on botanical and geographical origin, as well as on the extraction method used for analysis or preparation [69,70,71,72]. Major groups of constituents include phenolic acids and their esters, flavonoids, terpenoids, waxes, and other minor compounds, although their relative abundance differs markedly among propolis types.

Among the best-characterized redox-active constituents of propolis are caffeic acid derivatives and flavonoids, including CAPE, pinocembrin, galangin, chrysin, apigenin, quercetin, and naringenin [70,71,72,73]. These constituents contribute substantially to the antioxidant and redox-related properties of propolis, although their relative contribution depends on the chemical profile of the investigated preparation [71,72,73].

Propolis extracts frequently demonstrate substantial antioxidant activity in chemical and in vitro studies, although the magnitude of this activity depends strongly on extraction conditions and phenolic composition [69,70,71,72,73]. This pronounced compositional variability limits direct comparison between studies and highlights the need for chemically characterized and standardized preparations.

### 3.3. Royal Jelly

Royal jelly is a nutrient-rich secretion produced predominantly by the hypopharyngeal and mandibular glands of worker bees and used to nourish developing larvae and the queen. In contrast to polyphenol-rich products such as honey and propolis, royal jelly is characterized by a distinct protein–lipid-rich composition. Its principal constituents include major royal jelly proteins (MRJPs), carbohydrates, lipids, free amino acids, vitamins, minerals, and smaller amounts of phenolic and volatile compounds [74,75].

A characteristic bioactive constituent and widely used quality marker of royal jelly is 10-hydroxy-2-decenoic acid (10-HDA), an unsaturated medium-chain fatty acid that is considered one of its most distinctive chemical components [75]. Other biologically relevant constituents include additional fatty acids, MRJPs, and bioactive peptides such as apisimin, royalisin, and jelleins [75]. Together, these components contribute to the distinctive biological profile of royal jelly, which differs considerably from that of phenolic-dominated bee-derived products.

Protein- and peptide-derived components may also contribute to the anti-inflammatory activity of royal jelly. In experimental studies, major royal jelly protein 3 (MRJP3) and peptides derived from its C-terminal tandem penta-peptide repeats reduced the expression of pro-inflammatory mediators, including TNF-α and IL-6, in LPS-stimulated cells, with anti-inflammatory activity also demonstrated in vivo [76]. These findings indicate that the biological activity of royal jelly proteins and peptides may extend beyond their nutritional role, although the available evidence is not specific to neurodegenerative disease models.

Royal jelly and its isolated constituents have demonstrated antioxidant and anti-inflammatory activities in experimental studies. These effects appear to involve both lipid and protein components rather than direct radical scavenging alone [75]. In particular, royal jelly-derived fatty acids have been shown to modulate inflammatory signaling and reduce the production of selected pro-inflammatory mediators in cellular models [77]. Such findings support the concept that the redox-related effects of royal jelly may depend substantially on modulation of cellular signaling and inflammatory responses.

The composition and biological activity of royal jelly vary with biological, environmental, processing, and storage-related factors [75]. Most available evidence remains preclinical, and interpretation therefore requires consideration of product characterization, standardization, dose, and experimental model.

### 3.4. Bee Pollen and Bee Bread

Bee pollen consists of floral pollen collected by honeybees and mixed with nectar or honey and bee-derived secretions during collection and transport. After deposition in honeycomb cells and further processing within the hive, pollen undergoes biochemical and microbial transformations that result in the formation of bee bread [56]. These transformations modify its chemical composition and contribute to greater stability during storage.

Both bee pollen and bee bread are nutritionally complex matrices containing proteins, carbohydrates, lipids, vitamins, minerals, and numerous minor bioactive constituents [53,74]. Particularly relevant to their antioxidant and redox-related properties are phenolic acids, flavonoids, carotenoids, phytosterols, and unsaturated fatty acids. Their qualitative and quantitative composition is strongly influenced by botanical origin, geographical location, environmental conditions, and processing. Comparative analyses have also demonstrated substantial differences in phenolic and flavonoid content between pollen and bee bread, including samples originating from the same apiary [53,74].

The transformation of pollen into bee bread may alter the accessibility and recovery of several bioactive compounds. Experimental studies and in vitro digestion models indicate that bee bread can exhibit greater bioaccessibility of selected phenolic constituents than the corresponding pollen, although this effect depends on product composition and analytical conditions [56]. Both products have demonstrated antioxidant activity in chemical and in vitro assays, which is frequently associated with their phenolic and flavonoid content [53,56].

Their marked botanical and compositional variability, together with differences in extraction and analytical methodology, should therefore be considered when comparing studies of bee pollen and bee bread [56].

### 3.5. Bee Venom

Bee venom is a complex defensive secretion composed predominantly of peptides, proteins, enzymes, and low-molecular-weight constituents. Its biological profile differs fundamentally from that of polyphenol-rich bee-derived products such as honey or propolis because its principal activities are related to membrane disruption, neuroactivity, immune modulation, and inflammatory signaling rather than to a high content of classical antioxidant compounds [78].

The major bioactive component of bee venom is melittin, an amphipathic peptide with potent membrane-active properties. Other important constituents include apamin, phospholipase A2, adolapin, mast cell-degranulating peptide, and various biogenic amines and minor peptides [78,79]. These components differ markedly in their biological actions. Melittin and phospholipase A2 can exert pronounced cytolytic, inflammatory, or immunomodulatory effects, whereas apamin primarily acts as a neuroactive peptide. Consequently, biological effects observed for whole bee venom should not automatically be attributed to an individual constituent, and findings obtained with isolated components should not be interpreted as equivalent to administration of the complete venom.

Although bee venom is not a major source of phenolic antioxidants, experimental studies indicate that it and some of its constituents can influence redox homeostasis indirectly through modulation of inflammatory signaling, ROS generation, antioxidant defenses, and cellular stress responses [78]. Some in vitro studies have also reported measurable free-radical-scavenging activity of bee venom preparations [80]. However, such chemical antioxidant effects should be interpreted cautiously and distinguished from redox modulation occurring in biological systems. The overall response to bee venom is strongly dependent on concentration, exposure conditions, cellular context, and the specific venom component investigated.

The potential biological and neuroprotective effects of bee venom must also be considered together with its toxicity and allergenic potential. Bee venom exposure can induce local and systemic adverse reactions, including severe hypersensitivity and anaphylaxis, while individual components such as melittin may exert membrane-disruptive and cytotoxic effects at higher concentrations [78,79]. These safety concerns, together with dose dependence, variability in venom composition, and differences between whole-venom preparations and isolated constituents, represent major limitations to therapeutic translation. A clinically established therapeutic window for bee venom or its individual constituents in neurodegenerative disorders has not yet been defined and is likely to depend on the specific preparation, dose, route of administration, and target indication. This issue is particularly important for parenterally administered bee venom and apitherapy, which entail direct systemic exposure and associated risks of cytotoxicity and hypersensitivity, whereas orally consumed bee-derived products generally present a different systemic safety profile because exposure is shaped by gastrointestinal processing, absorption, and first-pass metabolism [78,79]. Therefore, bee venom should be regarded primarily as a biologically active and potentially redox-modulating mixture rather than as a conventional antioxidant product.

### 3.6. Beeswax

Beeswax is a lipid-rich structural material composed predominantly of long-chain esters, hydrocarbons, and fatty acids, with only minor amounts of phenolic compounds [53]. Compared with honey, propolis, bee pollen, and bee bread, it generally exhibits lower phenolic content and weaker antioxidant activity in chemical assays. Its relevance to neuroprotective and redox-modulating effects therefore appears limited, and its inclusion in the present review primarily reflects completeness and its chemical distinction from other bee-derived matrices.

### 3.7. Comparative Overview of Bee-Derived Products

Bee-derived products differ substantially in their chemical composition, predominant bioactive constituents, redox-related relevance, evidence base, and major limitations. These differences are summarized in Table 2.

Overall, bee-derived products should not be regarded as a uniform class of antioxidant preparations. Their biological relevance depends not only on chemical composition but also on bioaccessibility, systemic exposure, metabolism, and tissue-specific availability. These issues are considered in detail in Section 4.

## 4. Bioavailability, Biological Relevance, and Standardization of Bee-Derived Products and Constituents

### 4.1. From Chemical Antioxidant Activity to Biological Redox Modulation

Chemical antioxidant assays, including DPPH, ABTS, and FRAP, are useful for comparing the radical-scavenging and reducing capacity of bee-derived products under controlled experimental conditions. However, activity observed in such assays cannot be directly extrapolated to biological effects in vivo [53]. In biological systems, the effect of a given product or constituent depends on multiple factors, including its release from the biological matrix, absorption, metabolism, distribution, local concentration, and interaction with endogenous redox-regulatory systems. Moreover, ROS are not exclusively damaging molecules but also participate in physiological signaling, and their complete elimination would therefore not represent a desirable biological outcome [81].

Selected bee-derived products and their constituents can influence endogenous antioxidant defenses, inflammatory responses, and cellular stress signaling [12,15]. These effects may occur at concentrations at which direct stoichiometric scavenging of reactive species is unlikely to be the dominant mechanism. Thus, the biological activity of such compounds may depend more strongly on modulation of cellular redox homeostasis than on the antioxidant capacity measured in cell-free systems.

Consequently, high antioxidant capacity in a cell-free assay should not be interpreted as evidence of comparable activity in a biological system. The biological relevance of bee-derived products depends on whether their constituents, or the metabolites formed from them, reach concentrations sufficient to modify cellular redox processes without disrupting physiological ROS-dependent signaling [81].

### 4.2. Absorption, Metabolism, and Blood–Brain Barrier Relevance

The biological relevance of orally consumed bee-derived products depends on the extent to which their bioactive constituents are released from the original matrix, absorbed, metabolized, and distributed to target tissues. These processes vary substantially among different classes of compounds. Flavonoids, for example, may occur in free or conjugated forms and undergo extensive intestinal and hepatic metabolism, including glucuronidation, sulfation, and methylation. Consequently, the compounds circulating after oral intake may differ considerably from those initially identified in the chemical profile of honey, propolis, bee pollen, or other bee-derived products [82].

This distinction is particularly important when considering potential effects within the central nervous system (CNS). BBB penetration is compound-specific and cannot be inferred solely from antioxidant activity, lipophilicity, or the presence of a constituent in a bee-derived product. Experimental studies indicate that some isolated bee-product-associated compounds can reach or interact with the CNS. Pinocembrin, for example, has demonstrated uptake at the BBB in experimental models, whereas CAPE has been reported to cross the BBB in rats, although direct quantitative evidence of its brain distribution remains limited [83,84]. However, such findings concern administration of isolated compounds and should not be interpreted as evidence that consumption of honey or propolis results in comparable CNS exposure.

Similar caution is required for royal jelly constituents. Experimental studies have shown that 10-HDA can protect BBB integrity and influence neuroinflammatory processes, but evidence of an effect on the BBB does not necessarily demonstrate that substantial amounts of the compound itself cross the barrier [85]. More generally, biological effects observed in brain tissue after peripheral administration may result from direct CNS exposure, modulation of the BBB or cerebral vasculature, or indirect systemic mechanisms. Accordingly, attenuation of systemic inflammation, endothelial protection, or preservation of BBB integrity should not be interpreted as evidence that the investigated compound crosses the BBB or achieves pharmacologically relevant concentrations within the CNS.

Route of administration provides an additional source of heterogeneity. Gastrointestinal absorption and first-pass metabolism are particularly relevant to orally administered products such as honey, propolis, royal jelly, bee pollen, and bee bread, whereas these barriers are not directly applicable to bee venom administered by injection or bee venom acupuncture [86,87]. Therefore, pharmacokinetic findings should be interpreted in the context of both the investigated compound or preparation and its route of administration. Taken together, the available evidence supports CNS relevance for selected bee-derived constituents, but remains insufficient to assume comparable brain exposure for complex bee-derived products as a whole.

### 4.3. Bioaccessibility, Bioavailability, and Formulation Limitations

Limited bioavailability may substantially restrict the biological effects of several constituents present in bee-derived products, particularly after oral administration. The extent of systemic exposure depends on multiple factors, including release of bioactive compounds from the original matrix, aqueous solubility, chemical and enzymatic stability, intestinal absorption, and subsequent metabolism [82]. These limitations are compound-specific and should not be generalized to all bee-derived products. For example, poorly soluble flavonoid constituents may exhibit limited gastrointestinal absorption, whereas royal jelly proteins can undergo hydrolysis by gastrointestinal enzymes during digestion [88].

The distinction between bioaccessibility and bioavailability is particularly important. Bioaccessibility describes the fraction of a compound released from the food or biological matrix and potentially available for intestinal absorption, whereas bioavailability additionally requires absorption and systemic exposure [89]. In vitro gastrointestinal digestion studies indicate that bee bread can exhibit greater bioaccessibility of selected phenolic constituents than bee pollen, although the magnitude of this effect is compound- and sample-dependent [89]. However, increased bioaccessibility should not be interpreted automatically as evidence of greater systemic bioavailability or enhanced delivery to the CNS.

Formulation strategies are being investigated to improve the stability, solubility, and delivery of poorly bioavailable natural compounds and bee-derived preparations. In propolis research, for example, polymeric, lipid-based, and inorganic nanocarriers have been investigated to improve physicochemical properties and delivery of bioactive constituents [90]. Importantly, improved physicochemical properties or systemic exposure do not in themselves demonstrate enhanced BBB penetration or neuroprotective efficacy. Such claims require direct pharmacokinetic or tissue-distribution evidence [91].

This concern is illustrated by chrysin, for which human pharmacokinetic data indicate very low systemic exposure to the parent compound after oral administration, largely because of extensive presystemic metabolism [92]. Pharmacological effects observed with purified CAPE, pinocembrin, chrysin, or other constituents therefore cannot be assumed to occur to the same extent following consumption of propolis, honey, or other complex bee-derived matrices. Bioavailability should consequently be considered together with product composition, dose, formulation, route of administration, and the chemical form of the compound investigated.

### 4.4. Standardization and Reproducibility

A major challenge in the experimental and potential clinical use of bee-derived products is the substantial variability of their chemical composition. Botanical and geographical origin, season, processing, extraction procedure, and storage conditions can influence the qualitative and quantitative profile of bioactive constituents [15,54]. This variability complicates comparison between studies, particularly when chemically insufficiently characterized preparations are investigated under the same general designation, such as “propolis extract” or “bee pollen”.

Standardization should therefore involve more than quantification of a single bioactive compound. For highly heterogeneous products such as propolis, the selection of marker compounds should reflect the botanical origin and chemical profile of the investigated preparation rather than rely on a universal set of constituents [93]. Comparative studies have demonstrated substantial regional differences in propolis composition, including variation in phenolic acids and flavonoids [54,72]. Extraction procedures may introduce an additional source of variability because solvent type and extraction conditions affect the recovery of individual constituents and the measured antioxidant activity [71].

Reproducibility requires detailed characterization of both the raw material and the final preparation used experimentally. At minimum, studies should report the botanical and geographical origin where applicable, processing and extraction conditions, analytical characterization of major constituents, and the administered dose of the complete preparation. Where possible, quantitative information on selected marker compounds and batch-to-batch consistency should also be provided [93]. Chromatographic and spectrometric approaches can support chemical fingerprinting and comparison between preparations, but analytical methods themselves should be appropriately validated and consistently reported [54].

These considerations are particularly important when comparing mechanistic, preclinical, and clinical evidence. Results obtained with one chemically defined extract cannot necessarily be generalized to another preparation marketed or described under the same bee-product category. Improved chemical characterization, standardized extraction and reporting procedures, and appropriate quality-control criteria are therefore essential for determining whether observed biological effects are reproducible and attributable to a defined preparation rather than to uncontrolled compositional variability [54,93]. The principal factors affecting biological interpretation, bioavailability, and reproducibility of bee-derived products are summarized in Table 3.

## 5. Molecular Mechanisms Linking Bee-Derived Products and Constituents to Neuroprotection

Mechanistic studies indicate that the neuroprotective effects associated with bee-derived products involve several interconnected processes, including regulation of endogenous antioxidant responses, inflammatory signaling, mitochondrial function, apoptosis, and proteostasis. The evidence reviewed in this section encompasses whole bee-derived products, chemically defined extracts, isolated bioactive constituents, individual venom components, and synthetic or chemically modified derivatives. These categories are not pharmacologically interchangeable, and mechanistic findings should therefore be attributed only to the specific preparation or compound investigated [23]. Moreover, demonstration of biological activity in vitro or after peripheral administration does not by itself establish systemic bioavailability, direct CNS exposure, or BBB penetration; these pharmacokinetic distinctions are discussed in Section 4. Importantly, the mechanisms discussed below should not be interpreted as exclusively redox-dependent, and for some bee-derived products or constituents the molecular basis of the reported neuroprotective effects remains only partially characterized. Evidence derived from non-neuronal models or computational analyses is identified as such and should be interpreted as mechanistic or hypothesis-generating evidence rather than as direct evidence of neuroprotection in neuronal or disease-specific systems.

### 5.1. Activation of the Nrf2/ARE Antioxidant Response

Activation of the Nrf2/ARE pathway represents an important redox-related mechanism investigated for selected bee-derived constituents. CAPE can activate Nrf2 through mechanisms involving redox-sensitive modulation of Keap1 [95]. In HepG2 cells, CAPE-induced Nrf2 activation was associated with ERK signaling and increased expression of heme oxygenase-1, supporting an indirect antioxidant mechanism based on enhancement of endogenous cellular defenses rather than direct radical scavenging alone [96]. Although these models are non-neuronal, they provide mechanistic evidence that CAPE can enhance endogenous antioxidant signaling through distinct upstream mechanisms.

Evidence from neuronal models further supports the involvement of this pathway. Pinocembrin attenuated 6-OHDA-induced neuronal injury in SH-SY5Y cells through activation of the Nrf2/ARE pathway [97], while in an MPP+-induced model it increased heme oxygenase-1 expression through ERK1/2-dependent signaling [98]. Similarly, melittin activated the Keap1/Nrf2/HO-1 pathway in glutamate-stressed HT22 hippocampal cells, where this response was associated with reduced oxidative and inflammatory injury [99]. These findings suggest that chemically distinct bee-derived constituents may converge on Nrf2-associated signaling, although the upstream mechanisms responsible for pathway activation are not necessarily identical.

Royal jelly-associated fatty-acid derivatives have also been investigated in this context. The synthetic ester 4-hydroperoxy-2-decenoic acid ethyl ester (HPO-DAEE) protected SH-SY5Y cells against 6-OHDA-induced injury through activation of the Nrf2/ARE and eIF2α/ATF4 pathways [100]. This finding should, however, be distinguished from evidence obtained with native royal jelly or 10-HDA, because HPO-DAEE represents a chemically modified derivative rather than the natural product itself.

A similar distinction is required for CAPE derivatives. FA-97, a synthetic CAPE derivative, reduced oxidative stress-mediated neuronal apoptosis and cognitive impairment through activation of Nrf2/HO-1 signaling [101]. In addition, CAPE itself has demonstrated Nrf2/HO-1-dependent neuroprotective effects in an experimental model of Alzheimer’s disease [102]. Collectively, these studies support Nrf2-associated signaling as an important mechanistic link between selected bee-derived constituents and cellular resistance to oxidative stress, while also emphasizing that evidence obtained with synthetic derivatives should not be directly attributed to the corresponding natural bee product.

### 5.2. Inhibition of NF-κB-Dependent Neuroinflammation

NF-κB-dependent signaling represents another important mechanistic target of selected bee-derived constituents. CAPE can interfere with this pathway at the level of IκB kinase signaling, thereby limiting IκB degradation and subsequent NF-κB activation [103]. Notably, CAPE-mediated Nrf2 activation and inhibition of IκB kinase have been observed within the same experimental framework, illustrating that antioxidant and inflammatory signaling pathways may be modulated concurrently rather than independently [103].

Whole bee venom preparations have also demonstrated NF-κB-related anti-inflammatory effects in experimental models. In a lipopolysaccharide-induced model of neuroinflammation and memory impairment, bee venom reduced NF-κB pathway activation and attenuated associated inflammatory responses [104]. Similarly, in BV2 microglial cells, bee venom suppressed MyD88-dependent inflammatory signaling and reduced NF-κB nuclear translocation, accompanied by decreased expression of pro-inflammatory mediators including cyclooxygenase-2, TNF-α, IL-1β, and IL-6 [105]. These findings concern whole bee venom preparations and should not be attributed specifically to melittin or another individual venom constituent unless this has been demonstrated experimentally.

Among isolated bee venom components, apamin has been shown to modulate signaling involving CaMKII, ERK, p65, and STAT3 in MPP+-challenged dopaminergic neuronal models [106]. This effect was associated with reduced calcium-overload-related neurotoxicity and improved neuronal survival. Thus, whole bee venom and individual venom peptides may converge on NF-κB-associated signaling through distinct upstream mechanisms, although their broader cellular effects are not necessarily equivalent [106].

Anti-inflammatory activity has also been reported for protein- and peptide-derived components of royal jelly. MRJP3 and peptides derived from its C-terminal tandem penta-peptide repeats reduced TNF-α and IL-6 expression in LPS-stimulated THP-1 cells and attenuated inflammatory responses in vivo [76]. However, direct involvement of NF-κB signaling was not established in that study, and these findings should therefore be regarded as evidence of anti-inflammatory activity rather than as direct evidence of NF-κB inhibition or neuroprotection.

Suppression of NF-κB signaling may reduce the expression of inflammatory mediators that contribute to sustained microglial activation and neuronal injury [47]. However, these effects should be interpreted in the context of the specific experimental model and investigated agent, particularly because whole bee venom, isolated peptides, and purified bee-product-associated compounds are not mechanistically interchangeable.

### 5.3. Modulation of NLRP3 Inflammasome Activation

The NLRP3 inflammasome represents an important point of convergence between oxidative stress, mitochondrial dysfunction, and inflammatory signaling. Its activation is influenced by both transcriptional priming, including NF-κB-dependent expression of inflammasome components, and subsequent cellular stress signals associated with mitochondrial dysfunction and ROS generation [48]. Consequently, bee-derived compounds that affect redox homeostasis or inflammatory signaling may indirectly modify NLRP3 activity through more than one mechanism.

Experimental evidence supports such an interaction for selected polyphenolic compounds. In microglial SIM-A9 and neuronal SH-SY5Y cells, intracellular zinc deficiency increased ROS production and NLRP3 inflammasome-related responses, whereas caffeic acid derivatives, including CAPE, attenuated these effects [107]. Notably, this evidence derives from a cellular zinc-deficiency model and should not be interpreted as direct evidence of NLRP3 modulation in a specific neurodegenerative disease.

Apigenin has also been shown to interfere more directly with inflammasome assembly. In LPS-stimulated macrophages, apigenin inhibited caspase-1 activation and disrupted ASC oligomerization, thereby interfering with the assembly of a functional NLRP3 inflammasome [108]. In a rat model of spinal cord injury, apigenin promoted mitophagy, reduced ROS accumulation, suppressed NLRP3 inflammasome activation and pyroptosis, and improved functional recovery [109]. The latter finding is particularly relevant because mitochondrial quality control may influence inflammasome activation by limiting the accumulation of dysfunctional, ROS-generating mitochondria.

Inhibition of NLRP3 signaling ultimately reduces downstream caspase-1 activation and maturation of pro-inflammatory cytokines such as IL-1β [48]. However, the available evidence derives from different experimental contexts, including neuronal and microglial cell models, macrophages, and acute spinal cord injury [107,108,109]. These findings therefore support NLRP3 modulation as a plausible mechanistic link between selected bee-product-associated compounds and reduced inflammatory stress, but they do not establish a uniform effect across bee-derived products or neurodegenerative diseases.

### 5.4. Mitochondrial Protection and Quality Control

Mitochondrial dysfunction can amplify oxidative injury through disruption of membrane potential, excessive mitochondrial ROS generation, and impaired removal of damaged mitochondria [110]. Experimental evidence indicates that selected bee-derived products and associated compounds can influence several of these processes, although the available studies differ considerably in the experimental models and preparations used.

As discussed above, apigenin promoted mitophagy and suppressed ROS/NLRP3-associated inflammasome activation and pyroptosis in a rat model of spinal cord injury [109]. This finding illustrates a potential link between mitochondrial quality control and inflammatory signaling, although evidence from an acute injury model should not be extrapolated directly to chronic neurodegenerative diseases.

Direct effects on mitochondrial membrane integrity have also been reported for propolis preparations. In isolated rat liver mitochondria exposed to ferulenol, Algerian propolis extract restored the ferulenol-induced loss of mitochondrial membrane potential [111]. Although this finding supports a mitochondria-protective effect of the tested extract, the non-neuronal experimental system limits direct extrapolation to neuroprotection.

CAPE has additionally been associated with UCP2-dependent regulation of mitochondrial ROS production. In ARPE-19 retinal pigment epithelial cells, CAPE reduced basal and stress-induced ROS generation, whereas pharmacological inhibition or siRNA-mediated knockdown of UCP2 markedly diminished this protective effect [112]. The same study also demonstrated protection of retinal ganglion cells in vivo; however, the detailed UCP2-dependent mechanistic evidence was obtained primarily in ARPE-19 cells, and direct extrapolation to other CNS neuronal populations therefore remains limited.

Together, these findings suggest that mitochondrial effects of bee-derived products and constituents may involve preservation of membrane function, limitation of mitochondrial ROS production, and enhancement of mitochondrial quality control [109,111,112]. The available evidence, however, remains heterogeneous and is derived from distinct cellular and tissue models, which should be considered when evaluating its relevance to neurodegenerative disease.

### 5.5. Anti-Apoptotic and Pro-Survival Signaling

Modulation of apoptotic signaling represents another mechanism through which selected bee-derived products and constituents may protect cells exposed to oxidative or neurotoxic stress. Rather than acting through a single pathway, the available evidence indicates effects at different levels of the apoptotic response, including regulation of Bcl-2 family proteins and activation of intracellular pro-survival signaling [113,114].

Pinocembrin has been shown to influence the balance between pro- and anti-apoptotic proteins. In glutamate-challenged SH-SY5Y cells, pinocembrin increased cell viability, reduced LDH release and apoptosis, and decreased the Bax/Bcl-2 ratio, an effect associated primarily with reduced Bax expression rather than increased Bcl-2 expression [113]. These findings suggest that its protective effects involve modulation of mitochondrial apoptosis in addition to the redox-related mechanisms described above.

Whole bee venom has also demonstrated anti-apoptotic effects in neuronal cell models. In MPP+-treated SH-SY5Y cells, whole bee venom increased cell viability, reduced apoptotic cell death, increased Bcl-2 expression, and decreased Bax and cleaved PARP levels. Bee venom also prevented MPP+-induced suppression of Akt phosphorylation, whereas pharmacological inhibition of PI3K attenuated its neuroprotective effect, supporting involvement of the PI3K/Akt pathway [114]. Because the study investigated whole bee venom, these effects cannot be assigned to melittin, apamin, or another individual venom component without additional evidence.

CAPE appears to influence several stress-responsive signaling pathways associated with neuronal survival. In rat organotypic midbrain slice cultures, CAPE protected dopaminergic neurons through mechanisms involving p38 MAPK-dependent Nrf2 activation and induction of HO-1 and BDNF [115]. In acrolein-exposed HT22 hippocampal cells, CAPE, investigated alongside caffeic acid, attenuated ROS accumulation and GSH depletion and reduced neurotoxicity, with modulation of MAPK and Akt/GSK3β signaling implicated in these effects [116]. These findings indicate that CAPE can engage both antioxidant and pro-survival responses, although the role of individual MAPK pathways is context-dependent and should not be interpreted as uniformly anti-apoptotic.

Taken together, the available experimental evidence suggests that bee-derived products and constituents can influence neuronal survival by modifying the balance between apoptotic and pro-survival signaling. However, the specific pathways involved depend on the investigated agent, cellular stressor, and experimental model, and mechanistic findings obtained with isolated constituents should not be directly extrapolated to the corresponding whole bee-derived products.

### 5.6. Proteostasis, Protein Aggregation, and Amyloidogenic Processing

Disruption of proteostasis and accumulation of misfolded or aggregated proteins are central features of several neurodegenerative diseases [117]. Experimental studies indicate that selected bee-derived products and associated constituents may influence different components of protein homeostasis, including proteasomal degradation, pathological protein aggregation, and amyloidogenic processing.

In hSOD1G85R-expressing NSC34 motor neuron-like cells, whole bee venom restored proteasome activity and reduced the accumulation of ubiquitinated mutant SOD1 and misfolded SOD1 [118]. These findings suggest that modulation of intracellular protein clearance may contribute to the protective effects observed in this cellular model, although the evidence is limited to an experimental system expressing mutant SOD1.

Protein aggregation has also been targeted using engineered materials derived from propolis polyphenols. Propolis polyphenol-based nanosheets inhibited α-synuclein amyloid aggregation and redirected fibril assembly toward less cytotoxic off-pathway oligomeric species [119]. Importantly, these findings concern a specifically engineered polyphenol-based nanomaterial and should not be interpreted as evidence that unmodified propolis exerts an equivalent anti-aggregation effect.

Purified royal jelly-derived peptide fractions have been investigated in relation to amyloidogenic processing. In N2a/APP695swe cells, these peptides downregulated BACE1 and reduced extracellular Aβ1–40 and Aβ1–42 production [120]. An in silico screening study additionally identified several bee-product-associated phytochemicals, including rutin, 3,4-dicaffeoylquinic acid, nemorosone, and luteolin, as candidate high-affinity BACE1 ligands [121]. However, these computational predictions should be regarded as hypothesis-generating and require experimental validation before direct BACE1 inhibition or neuroprotective efficacy can be inferred.

Collectively, these findings indicate that selected bee-derived products, derived fractions, and associated compounds may influence protein homeostasis at several distinct levels, including proteasomal clearance of misfolded proteins and modulation of pathological protein aggregation [118,119], as well as amyloidogenic processing [120,121]. Nevertheless, the available evidence is predominantly preclinical and includes cellular models, engineered materials, and computational predictions. The strength of mechanistic evidence therefore varies considerably among the investigated agents and should not be interpreted as demonstrating a common anti-aggregation effect of bee-derived products as a whole. Representative mechanistic evidence across these domains is summarized in Table 4.

**Figure 2:**
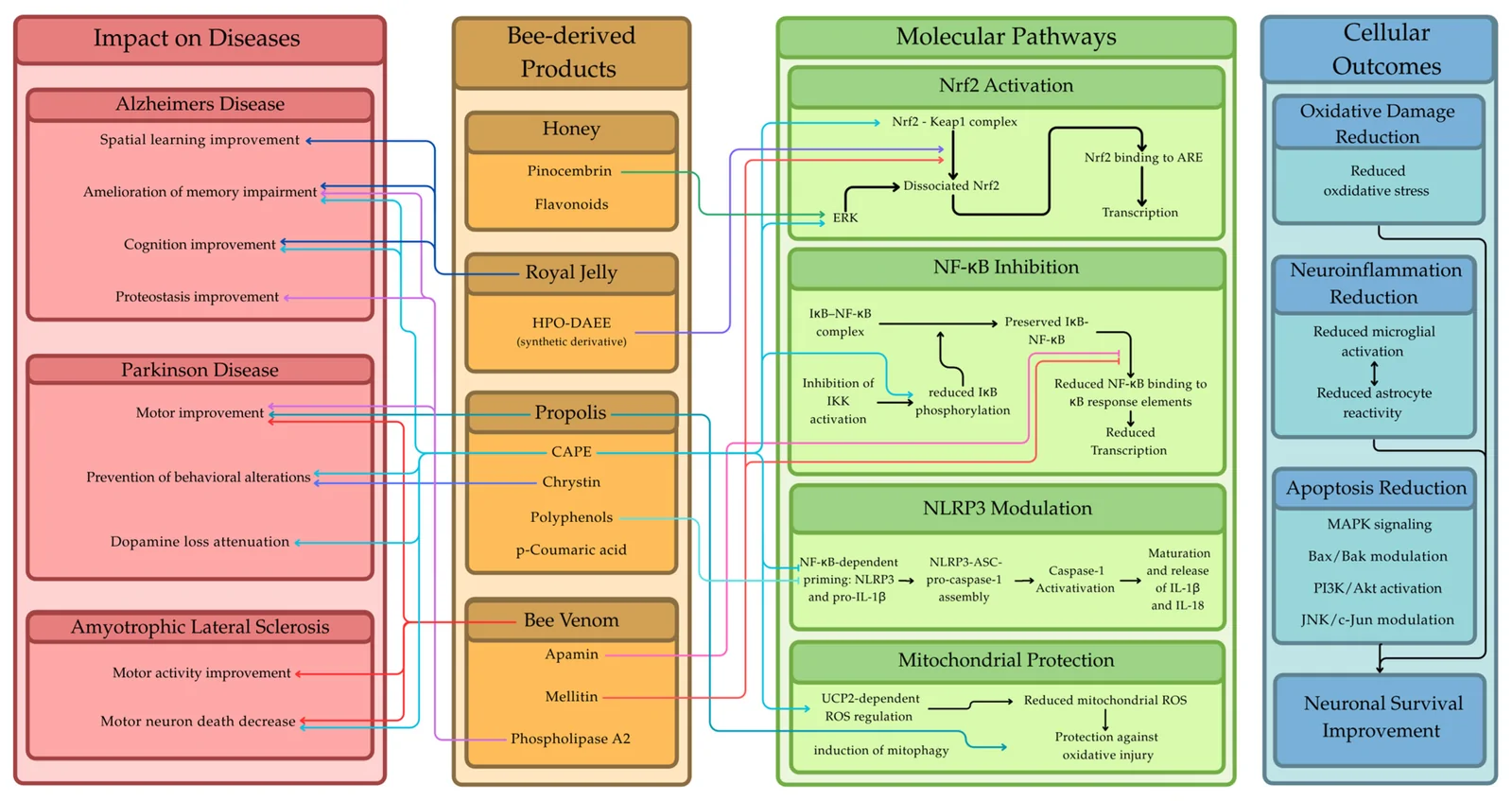
Integrated overview of representative bee-derived products and associated constituents, molecular pathways, cellular outcomes, and disease-related effects relevant to neuroprotection. The central panel presents selected bee-derived products and representative associated constituents. Connections to the right summarize experimentally reported associations with molecular pathways relevant to neuroprotection, including Nrf2 activation, NF-κB inhibition, NLRP3 inflammasome modulation, and mitochondrial protection, together with representative downstream cellular outcomes. Connections to the left summarize selected beneficial effects reported in experimental models of Alzheimer’s disease, Parkinson’s disease, and amyotrophic lateral sclerosis. The figure integrates findings from heterogeneous experimental studies; individual connections may therefore originate from different experimental models and should not be interpreted as a single continuous causal pathway from a given constituent to a disease-related outcome. Moreover, the depicted associations do not imply that every molecular pathway, cellular outcome, or disease-related effect has been demonstrated for each corresponding whole bee-derived product. Findings obtained with whole products, isolated constituents, individual venom components, synthetic derivatives, or engineered preparations should not be regarded as pharmacologically equivalent. HPO-DAEE represents a synthetic royal jelly-related derivative rather than a naturally occurring royal jelly constituent. Arrowheads indicate the direction of the depicted processes or associations. Black arrows represent simplified relationships within the molecular-pathway and cellular-outcome panels, whereas colored connecting arrows are used to facilitate visual tracking of associations between bee-derived products or constituents and the depicted pathways or disease-related effects; the colors do not indicate equivalent levels of evidence or distinct classes of molecular regulation. Abbreviations: ARE, antioxidant response element; ASC, apoptosis-associated speck-like protein containing a caspase activation and recruitment domain; Akt, protein kinase B; Bax, Bcl-2-associated X protein; Bcl-2, B-cell lymphoma 2; CAPE, caffeic acid phenethyl ester; ERK, extracellular signal-regulated kinase; HPO-DAEE, 4-hydroperoxy-2-decenoic acid ethyl ester; IκB, inhibitor of nuclear factor κB; IKK, IκB kinase; IL, interleukin; JNK, c-Jun N-terminal kinase; Keap1, Kelch-like ECH-associated protein 1; MAPK, mitogen-activated protein kinase; NF-κB, nuclear factor κB; NLRP3, NLR family pyrin domain containing 3; Nrf2, nuclear factor erythroid 2-related factor 2; PI3K, phosphoinositide 3-kinase; ROS, reactive oxygen species; UCP2, uncoupling protein 2.

The principal relationships between representative bee-derived products and associated constituents, molecular pathways relevant to neuroprotection, downstream cellular outcomes, and disease-related effects are summarized schematically in Figure 2. The figure integrates mechanistic and disease-specific findings from heterogeneous experimental studies and is intended to provide an overview of reported associations rather than a single causal sequence linking individual constituents, pathways, and disease outcomes.

## 6. Disease-Specific Evidence for Bee-Derived Products and Constituents in Neurodegenerative Disorders

The mechanistic findings summarized above have been investigated across a range of experimental models relevant to neurodegenerative diseases. However, the strength and translational relevance of the available evidence vary considerably according to the investigated product or constituent, experimental model, route of administration, and outcome assessed [23]. Most available studies remain preclinical, and findings obtained with isolated compounds, individual bee venom components, or other defined preparations should not be interpreted as equivalent to the effects of the corresponding whole bee-derived product.

### 6.1. Alzheimer’s Disease

Alzheimer’s disease (AD) involves progressive synaptic and neuronal dysfunction associated with amyloid-β (Aβ) and tau pathology, together with mitochondrial impairment, redox dysregulation, and neuroinflammation [122]. Bee-derived products and constituents have therefore been investigated in experimental models addressing cognitive function, amyloid-related pathology, oxidative stress, and inflammatory responses.

#### 6.1.1. CAPE

CAPE has demonstrated protective effects across several experimental models relevant to AD and cognitive dysfunction. In Aβ1–42 oligomer-injected mice, CAPE attenuated memory impairment and hippocampal neuronal loss, with activation of Nrf2/HO-1 signaling, modulation of GSK3β, and reduced glial activation [102]. In intracerebroventricular streptozotocin models, CAPE improved cognitive outcomes, increased brain GSH, limited lipid peroxidation, and showed evidence of PI3K-dependent nitric oxide signaling [123,124]. Beneficial behavioral and physiological effects have also been reported in tau-expressing *Drosophila melanogaster* and a scopolamine-induced mouse model [125]. Collectively, these findings support redox- and survival-signaling effects of isolated CAPE across heterogeneous experimental systems, although these models reproduce only selected aspects of human AD.

#### 6.1.2. Bee Venom Phospholipase A2 and Bee Venom

Bee venom phospholipase A2 (bvPLA2) has shown neuroprotective and immunomodulatory effects in several AD-related mouse models. In Tg2576 mice, bvPLA2 improved memory and reduced amyloidogenic and neuroinflammatory alterations, with inhibition of STAT3-related signaling [126]. In 3xTg-AD mice, beneficial effects were associated with increased regulatory T-cell responses and reduced neuroinflammation, and depletion of regulatory T cells markedly diminished the protective response [127]. In a related Aβ-vaccination model, bvPLA2 attenuated vaccination-associated inflammation while reducing Aβ burden and cognitive deficits [128].

In an LPS-induced AD-like model, bvPLA2 improved memory-related outcomes and reduced amyloidogenic and inflammatory changes, including NF-κB-associated signaling [129]. Whole bee venom produced broadly similar behavioral and inflammatory effects in an LPS-associated model [104]. However, purified bvPLA2 and whole bee venom represent pharmacologically distinct interventions; evidence obtained with one should not be extrapolated to the other, and whole-venom effects cannot be assigned to a specific constituent without direct experimental evidence.

#### 6.1.3. Royal Jelly

Royal jelly has shown potentially beneficial effects across several mammalian models of cognitive impairment and AD-related pathology. In streptozotocin-based rat models, royal jelly improved learning and memory and was associated with reduced hippocampal neurodegeneration and oxidative stress [130,131]. Beneficial effects on cognition, oxidative and inflammatory alterations, and neuronal injury were also reported in an okadaic acid model [132]. In APP/PS1 mice, whole royal jelly reduced cognitive deficits, Aβ accumulation, and neuronal apoptosis, with involvement of cAMP/PKA/CREB/BDNF signaling [133]. Additional studies in cholesterol/copper- and Aβ1–40-based models reported attenuation of amyloid-related pathology and hippocampal oxidative stress [134,135]. Importantly, these findings concern whole royal jelly preparations rather than isolated 10-HDA.

Complementary evidence has been obtained in an Aβ-expressing *Caenorhabditis elegans* model, in which royal jelly and enzyme-treated royal jelly delayed Aβ-associated paralysis and improved proteostasis through DAF-16-dependent mechanisms [136]. These findings broaden the mechanistic evidence base but should be regarded as supportive evidence from an invertebrate proteotoxicity model rather than as equivalent to efficacy in mammalian AD.

Overall, the available AD evidence remains entirely preclinical and derives from heterogeneous transgenic, chemically induced, Aβ-administration, and invertebrate models. Although CAPE, bvPLA2, whole bee venom, and royal jelly have shown potentially beneficial effects, differences in preparation, dose, route of administration, and experimental model limit direct comparison and do not establish therapeutic efficacy in human AD. Key studies are summarized in Table 5.

### 6.2. Parkinson’s Disease

Parkinson’s disease (PD) is characterized by progressive degeneration of nigrostriatal dopaminergic neurons and pathological α-synuclein accumulation, accompanied by mitochondrial and redox dysfunction, impaired proteostasis, and neuroinflammation [137,138]. Bee-derived products and constituents have therefore been investigated in toxin-based, genetic, and other experimental models of dopaminergic neurodegeneration, as well as in a limited number of clinical studies.

#### 6.2.1. Non-Venom Bee-Derived Products and Constituents

CAPE has demonstrated neuroprotective effects across several experimental models of dopaminergic injury. In MPTP- and 6-OHDA-based models, CAPE attenuated dopaminergic neuronal loss, dopamine depletion, free-radical generation, and mitochondrial dysfunction [84,139,140]. Additional studies involving organotypic midbrain cultures and in vivo dopaminergic injury showed p38 MAPK-dependent Nrf2 activation, induction of HO-1 and BDNF, reduced microglial/macrophage activation, and improved behavioral outcomes [115]. Collectively, these findings support redox-, mitochondrial-, and survival-signaling effects of isolated CAPE, but should not be extrapolated directly to whole propolis.

Evidence for propolis and propolis-associated interventions is more heterogeneous. Ethanolic propolis extract improved motor performance, antioxidant-related parameters, and lifespan in a *PINK1B9 Drosophila melanogaster* model [141], whereas the isolated flavonoid chrysin attenuated behavioral and neuroinflammatory abnormalities in 6-OHDA-treated mice [142]. A liquiritigenin-rich Brazilian red propolis extract reduced L-DOPA-induced dyskinesia in hemiparkinsonian rats [143], although this model primarily addressed treatment-related dyskinesia rather than prevention of primary dopaminergic neurodegeneration. Propolis polyphenol-based nanosheets also produced antioxidant and neuroprotective effects in rotenone-based models [144]; however, this engineered formulation should not be considered equivalent to conventional propolis extracts.

The royal jelly-associated fatty acid 10-hydroxy-2-decenoic acid (10-HDA) has also shown neuroprotective activity in PD-related experimental systems. Isolated 10-HDA promoted autophagy through Beclin-1/mTOR- and SIRT1-associated mechanisms in cellular, mouse, and fly models [145]. These findings concern the isolated constituent and should not be interpreted as equivalent evidence for whole royal jelly.

#### 6.2.2. Bee Venom and Venom-Derived Constituents

Bee venom-based interventions have shown neuroprotective, redox-modulating, and anti-inflammatory effects in several toxin-induced PD models. In rotenone-treated mice, bee venom acupuncture and whole bee venom improved motor or neurochemical outcomes and attenuated oxidative, nitrosative, inflammatory, and apoptotic alterations [146,147]. In MPTP-treated mice, whole bee venom reduced dopaminergic neuronal degeneration and microglial activation, with increased regulatory T-cell responses implicated in the protective effect [148]. These findings suggest that whole-venom interventions may engage both redox-related and neuroimmune mechanisms, although the route and mode of administration differ substantially among studies.

Individual venom constituents show distinct biological profiles and should be considered separately from whole bee venom. In a chronic MPTP/probenecid model, apamin reproduced only part of the neuroprotective effect of whole bee venom, indicating that a single peptide does not account for the activity of the complete preparation [149]. Purified bvPLA2 improved motor dysfunction and reduced α-synuclein-associated pathology and microglial activation in A53T α-synuclein transgenic mice [150], while a standardized GMP-produced bvPLA2 preparation provided dose-dependent protection in MPTP-treated mice and induced regulatory T-cell responses [151]. These findings reinforce the pharmacological distinction between whole bee venom, apamin, and purified bvPLA2.

#### 6.2.3. Clinical Evidence for Bee Venom-Based Interventions

Clinical evidence for bee venom-based interventions in PD remains limited and inconsistent. In a randomized double-blind placebo-controlled trial involving patients with moderately advanced PD, monthly subcutaneous bee venom injections administered for 11 months did not significantly improve motor symptoms or imaging measures of disease progression compared with placebo [152]. This negative result is particularly important when interpreting the numerous beneficial effects reported in animal models.

Other clinical studies evaluated bee venom acupuncture, generally as part of a combined acupuncture-based intervention and alongside continued standard pharmacological treatment. A small prospective open-label study reported improvements in gait, activities of daily living, quality of life, and motor symptoms [153], while a subsequent three-arm randomized controlled trial also reported improvements in selected clinical outcomes [154]. However, because these interventions combined conventional acupuncture with bee venom acupuncture, the bee venom-specific contribution cannot be isolated. A later randomized neuroimaging study demonstrated changes in regional cerebral perfusion, whereas the increase in striatal dopamine transporter binding was not significantly different between groups [155]; these findings should therefore be regarded as exploratory mechanistic evidence rather than confirmation of disease modification.

Overall, the PD evidence spans extensive but heterogeneous preclinical studies and a small number of clinical investigations. Although several bee-derived products and constituents have shown potentially beneficial effects experimentally, human evidence remains limited, and the available studies involve pharmacologically distinct interventions ranging from subcutaneous whole bee venom to multimodal acupuncture-based treatment. These approaches should not be considered equivalent, and current evidence does not establish clinical efficacy of bee-derived interventions in PD. Key preclinical and clinical findings are summarized in Table 6.

### 6.3. Amyotrophic Lateral Sclerosis

Amyotrophic lateral sclerosis (ALS) is characterized by progressive degeneration of upper and lower motor neurons and involves redox and mitochondrial dysfunction, neuroinflammation, and impaired proteostasis [159]. Most available evidence for bee-derived interventions derives from mutant *SOD1* models; because *SOD1* mutations account for only a subset of familial ALS, these models reproduce only part of the genetic and pathological heterogeneity of human disease.

Bee venom-based interventions have been evaluated mainly in symptomatic hSOD1G93A mice. Subcutaneous bee venom administered bilaterally at the ST36 acupoint improved motor performance and prolonged survival, accompanied by reduced microglial activation and phospho-p38 MAPK expression, lower caspase-3 activity, and preservation of mitochondrial ultrastructure [160]. In a related study, bee venom acupuncture at ST36 improved motor function and motor neuron survival and suppressed TLR4-, CD14-, and TNF-α-associated inflammatory signaling; direct comparison with intraperitoneal bee venom demonstrated route- and site-dependent differences in central and peripheral responses [161]. These findings support neuroinflammatory, apoptotic, and mitochondrial effects, but bee venom acupuncture and systemic bee venom administration should not be regarded as equivalent interventions. Notably, oxidative stress itself was not directly measured in the first study [160].

CAPE has also been evaluated in symptomatic hSOD1G93A mice. Oral administration initiated after disease onset prolonged post-onset survival and overall lifespan and was associated with greater motor neuron preservation, reduced microglial and astrocytic activation, and lower phospho-p38 MAPK levels in the spinal cord [162]. Because CAPE is an isolated propolis-associated constituent, these findings should not be extrapolated directly to whole propolis. The post-onset treatment design provides somewhat greater translational relevance than purely preventive approaches, although the evidence remains limited to an SOD1-based model.

Cellular mutant-SOD1 models provide additional evidence for propolis preparations and associated phenolic constituents. In N2a cells, Brazilian green propolis extract reduced mutant SOD1-associated neurotoxicity and intracellular SOD1 aggregation, with autophagy implicated in these effects; kaempferide and kaempferol were identified as active constituents, both reducing mitochondrial superoxide generation, while kaempferol was additionally associated with AMPK/mTOR-dependent autophagy [163]. In a related model, p-coumaric acid attenuated mutant SOD1-associated toxicity through activation of autophagy [164]. These findings primarily support modulation of autophagy and proteostatic clearance rather than demonstrating that direct radical scavenging accounts for the observed neuroprotection.

Complementary evidence has been obtained in a Ubiquilin-knockdown *Drosophila melanogaster* model relevant to ALS/frontotemporal dementia-associated proteostatic dysfunction. Coffee honey from *Apis cerana* and isolated melittin from *Apis dorsata* both improved locomotor defects, but only coffee honey significantly improved learning, partially rescued neuromuscular-junction abnormalities, and reduced ROS accumulation in larval brains [165]. The effects of whole honey and isolated melittin should therefore remain clearly distinguished and should not be combined into a single mechanistic conclusion.

Overall, the ALS evidence remains entirely preclinical and derives from heterogeneous SOD1-based cellular and animal models together with an ALS/FTD-related invertebrate model. These studies support potential effects on neuroinflammation, mitochondrial integrity, apoptosis, autophagy/proteostasis, and oxidative stress, but no clinical evidence currently establishes therapeutic efficacy of bee-derived interventions in ALS. Key experimental findings are summarized in Table 7.

### 6.4. Other Neurological Models Relevant to Neurodegeneration

Complementary evidence for neuroprotective effects of bee-derived products and constituents has been obtained in vascular, ischemic, aging-related, and retinal models. Although these experimental systems do not directly reproduce chronic neurodegenerative diseases, they provide additional information on neuronal vulnerability, oxidative injury, vascular dysfunction, and cognitive impairment.

In vascular and ischemic models, Malaysian Tualang honey reduced hippocampal CA1 neuronal loss and preserved tissue morphology after chronic cerebral hypoperfusion in rats [166], although the molecular mechanism was not directly investigated. Brazilian green propolis reduced cerebral infarct volume and brain swelling after permanent middle cerebral artery occlusion in mice [167]; complementary antioxidant assays suggested a potential contribution of reduced oxidative injury, although this mechanistic link remains inferential. Royal jelly hydrolysate (RJH) also showed cytoprotective activity in H_2_O_2_- and glutamate-stressed SH-SY5Y cells and improved spatial memory with reduced hippocampal acetylcholinesterase activity in a rat model of vascular dementia [168]. Importantly, these findings concern a hydrolyzed royal jelly preparation and should not be extrapolated directly to untreated whole royal jelly.

CAPE has additionally demonstrated protective effects in aging- and retina-related models. In aged Sprague–Dawley rats, chronic CAPE administration reduced brain and cerebellar MDA levels, improved selected antioxidant defense parameters, and attenuated age-associated ultrastructural alterations [169]. In H_2_O_2_-stressed photoreceptor-derived cells and retinal light-stress models, CAPE reduced cellular injury, increased HO-1 expression, reduced NF-κB nuclear translocation, and improved retinal functional responses [170]. These findings support redox- and stress-modulating activity of CAPE in neural tissues, but physiological aging and retinal injury should not be regarded as direct models of AD, PD, or ALS.

Overall, these complementary models broaden the evidence for neuroprotective and redox-modulating activity of selected bee-derived products and constituents, but their heterogeneous and predominantly vascular, acute, or non-disease-specific nature limits direct extrapolation to chronic neurodegenerative disorders. They should therefore be regarded as supportive mechanistic evidence rather than as evidence of disease-specific therapeutic efficacy. Key findings are summarized in Table 8.

### 6.5. Comparative Synthesis of Disease-Specific Evidence

Across the major neurodegenerative diseases considered in this review, the maturity of the evidence differs substantially. For AD, the available evidence remains entirely preclinical and derives from heterogeneous transgenic, chemically induced, and amyloid-related models. PD has the broadest translational evidence base, including extensive preclinical studies and a limited number of clinical investigations; however, the human findings remain inconsistent and do not establish therapeutic efficacy. Evidence for ALS is more limited and remains entirely preclinical, relying predominantly on mutant SOD1-based models. Thus, despite recurrent neuroprotective and redox-related effects across experimental systems, the overall evidence remains insufficient to support clinical efficacy of bee-derived interventions in any of these disorders.

## 7. Therapeutic Potential, Safety, Translational Limitations, and Future Perspectives

The evidence summarized in the preceding sections indicates that selected bee-derived products and associated constituents can modulate multiple biological processes relevant to neurodegeneration, including redox homeostasis, inflammatory signaling, mitochondrial function, cell survival, and proteostasis [23]. However, the available evidence is heterogeneous and predominantly preclinical, and it encompasses whole bee-derived products, chemically defined extracts, isolated constituents, venom-derived components, synthetic derivatives, and engineered formulations. Their therapeutic relevance therefore depends not only on biological activity but also on product characterization, clinically achievable exposure, safety, route of administration, and the strength of disease-specific evidence.

### 7.1. Therapeutic Potential and Clinical Positioning

The therapeutic potential of bee-derived products should be considered at the level of individual, chemically and pharmacologically defined interventions rather than as a property of a uniform therapeutic class. Whole products such as honey, propolis, royal jelly, and bee venom differ fundamentally from isolated constituents such as CAPE, pinocembrin, 10-HDA, apamin, or bvPLA2, and beneficial effects demonstrated for one preparation cannot be extrapolated automatically to another. Accordingly, the most realistic near-term value of bee-derived materials may lie in providing bioactive molecules, molecular scaffolds, or well-characterized biological preparations for further pharmacological development. Natural products have historically represented an important source of drug leads, but successful translation generally requires isolation or definition of active agents, characterization of pharmacological properties, optimization when necessary, and rigorous evaluation of efficacy and safety [171].

The possibility of developing standardized whole bee-derived products should not be excluded, but such preparations would require reproducible chemical characterization and evidence that biologically relevant exposure can be achieved under clinically applicable conditions. Conversely, neuroprotective effects observed after administration of an isolated constituent at an experimental dose do not demonstrate that consumption or administration of the corresponding natural product will produce comparable systemic or central nervous system exposure. Given the predominantly preclinical evidence summarized above and the limited human data, bee-derived products and their constituents should currently be regarded as investigational candidates rather than established treatments for neurodegenerative diseases.

### 7.2. Safety Considerations

Safety is a critical consideration in the therapeutic development of bee-derived products, particularly because neurodegenerative diseases are chronic conditions that would generally require repeated or prolonged treatment. Safety profiles cannot be generalized across whole bee products, isolated constituents, venom-derived components, and engineered formulations, because these interventions differ substantially in composition, pharmacological potency, dose, and route of administration [78].

Hypersensitivity represents one of the most important safety concerns. Bee venom administration can cause local and systemic allergic reactions, including potentially severe anaphylactic responses, and adverse events have been reported across different forms of bee venom therapy [172]. Allergic reactions are not restricted to venom: honey, royal jelly, bee pollen, bee bread, propolis, and other hive products can also provoke IgE-mediated or other hypersensitivity reactions in susceptible individuals [173]. Consequently, tolerability observed in short-term experimental models cannot be assumed to predict safety during repeated exposure in humans.

Dose and route of administration are additional determinants of risk. Oral consumption of honey, propolis, royal jelly, bee pollen, or bee bread is pharmacologically distinct from administration of purified constituents at experimental doses or from parenteral exposure to whole bee venom and its isolated components. Bee venom contains highly bioactive peptides and enzymes, and membrane-active constituents such as melittin may exert cytotoxic effects at sufficiently high concentrations; adverse effects are therefore strongly dependent on dose, exposure conditions, and administration route [78,87]. Accordingly, safety data obtained for whole venom cannot be transferred directly to purified bvPLA2 or apamin, and findings from isolated constituents or engineered delivery systems require preparation-specific evaluation.

Long-term safety remains insufficiently characterized for most bee-derived interventions investigated in neurodegenerative disease. Future development should therefore include preparation-specific dose-escalation and repeated-dose studies, systematic monitoring of immunogenicity and adverse events, and evaluation of clinically relevant routes and exposure levels before large efficacy trials are undertaken. Evidence of biological activity or natural origin should not be regarded as evidence of therapeutic safety.

### 7.3. Translational Limitations and Clinical Evidence

A major challenge in translating the experimental effects of bee-derived products into clinically useful interventions is the substantial heterogeneity of the investigated preparations. Complex products may differ in botanical origin, chemical composition, extraction procedure, and concentration of bioactive constituents, making comparison and reproduction of experimental findings difficult. This problem is particularly evident for propolis, for which substantial compositional variability and the need for analytical harmonization and chemical authentication have been emphasized [54,93]. In addition, many experimental studies employ isolated compounds, purified venom constituents, or specifically engineered formulations at doses and routes of administration that may not reproduce the exposure achievable with the corresponding natural product.

Limitations of the experimental models themselves represent an additional barrier to translation. Much of the disease-specific evidence summarized in this review derives from toxin-induced, transgenic, chemically induced, or other reductionist models that reproduce selected pathological mechanisms but do not fully recapitulate the chronic, age-dependent, and multifactorial nature of human neurodegenerative diseases. Animal models remain highly valuable for mechanistic investigation, but their ability to predict therapeutic efficacy in patients is limited, and successful effects in a single experimental model should therefore not be regarded as sufficient evidence for clinical development [174].

Human evidence remains sparse and is concentrated primarily on bee venom-based interventions in Parkinson’s disease. A randomized double-blind placebo-controlled trial of monthly subcutaneous bee venom injections did not demonstrate a clear symptomatic or disease-modifying benefit [152]. More favorable clinical signals have been reported in small studies evaluating acupuncture combined with bee venom acupuncture [153,154], while a subsequent randomized neuroimaging study provided exploratory evidence of treatment-associated changes in cerebral physiology [155]. These studies investigated multimodal acupuncture-based interventions and therefore do not allow the specific contribution of bee venom to be isolated. Comparable clinical evidence supporting therapeutic efficacy in AD or ALS is currently lacking.

Consequently, future clinical development should focus on clearly defined candidate interventions rather than on bee-derived products as a broad therapeutic category. Before efficacy trials are undertaken, candidate preparations should undergo reproducible chemical characterization, dose and route optimization, pharmacokinetic and safety evaluation, and, where feasible, demonstration of target engagement. Subsequent clinical studies should be adequately powered and use disease-relevant clinical and biomarker outcomes. At present, the available evidence supports continued translational investigation but does not justify established clinical use of bee-derived interventions for neurodegenerative diseases.

### 7.4. Future Perspectives

Future research should prioritize a limited number of well-defined candidate interventions rather than continue to expand the number of poorly characterized bee-derived preparations. Whole products intended for further development should be chemically fingerprinted, quantitatively characterized for relevant marker compounds, and evaluated for batch-to-batch reproducibility, whereas isolated constituents should be studied using dose ranges that permit meaningful pharmacokinetic and pharmacodynamic interpretation [93,171]. Direct comparisons between whole products and their proposed active constituents would be particularly informative, because such experiments could establish whether biological activity is attributable to a dominant compound, interactions among multiple constituents, or matrix-dependent effects.

Preclinical development should also move beyond reliance on acute toxin-based and single-pathway models. Promising candidates should be evaluated across complementary systems that better represent chronic, age-related, and genetically heterogeneous neurodegeneration, with incorporation of dose–response relationships, clinically relevant exposure, and molecular target engagement [174]. Human induced pluripotent stem cell-derived neurons, glial co-cultures, and three-dimensional cerebral organoid systems may provide useful complementary platforms for testing candidate compounds in human disease-relevant cellular contexts, although these models also have important limitations and should complement rather than replace animal and clinical studies [175].

Candidates that demonstrate reproducible activity across chemically defined preparations and complementary experimental models should progress through a staged translational pathway including safety assessment, pharmacokinetic characterization, dose selection, and early demonstration of target engagement before adequately powered efficacy trials are undertaken. Biomarker-supported study designs may be particularly valuable for distinguishing biological target engagement from nonspecific symptomatic effects. Advanced delivery systems may improve the stability or exposure of selected poorly bioavailable compounds, but formulation strategies should be developed only when they address a demonstrated pharmacokinetic limitation and should not be assumed to confer improved CNS delivery or therapeutic efficacy without direct evidence. This stepwise approach would help distinguish pharmacologically credible candidates from the much broader group of bee-derived products for which biological activity has been demonstrated only under limited experimental conditions.

## 8. Conclusions

Bee-derived products comprise a chemically and pharmacologically heterogeneous source of bioactive compounds with potential relevance to neurodegenerative disorders. The reported biological effects should not be assumed to arise exclusively from redox modulation, particularly where the causal molecular mechanisms have not been directly established. Available evidence indicates that selected products and constituents can influence redox homeostasis, neuroinflammatory signaling, mitochondrial function, cell survival, and proteostasis, extending their biological activity beyond direct antioxidant effects. However, most findings remain preclinical and derive from whole products, specific extracts, isolated constituents, venom-derived components, synthetic derivatives, or engineered formulations that should not be considered pharmacologically equivalent. Clinical evidence is sparse and currently insufficient to establish therapeutic efficacy in neurodegenerative diseases. Accordingly, bee-derived products should not be regarded as a uniform therapeutic class; their principal current value lies in providing bioactive compounds and well-defined biological preparations for further pharmacological investigation. Translation will require reproducible preparations, appropriate pharmacokinetic and safety characterization, and substantially stronger human evidence. Until such evidence becomes available, their application in neurodegenerative disease should be considered investigational rather than therapeutic.

## Figures and Tables

**Table 1:** Representative honey types and major factors associated with their antioxidant properties.

Honey Type or Category	Characteristic Compositional Features	General Pattern in Chemical Antioxidant Assays	Key Considerations	References
Light blossom honeys (e.g., acacia, rapeseed, citrus)	Generally lower concentrations of phenolic compounds and flavonoids compared with many darker honeys	Frequently lower antioxidant capacity in comparative chemical assays	Considerable variability exists among samples; botanical origin, geographical origin, and processing conditions may substantially affect the chemical profile	[57,60,66,67]
Intermediate blossom honeys (e.g., linden, sunflower, thyme)	Variable phenolic acid and flavonoid profiles depending on floral source	Moderate to relatively high antioxidant capacity has been reported for selected monofloral honeys	Antioxidant properties cannot be predicted solely from the declared floral origin and should be interpreted together with compositional analysis	[67,68]
Dark blossom honeys (e.g., buckwheat, heather, chestnut)	Frequently enriched in phenolic compounds, including phenolic acids, flavonoids, and tannin-related constituents	Often exhibit comparatively high antioxidant capacity and reducing activity	The association between darker color, higher phenolic content, and greater antioxidant capacity is common but not universal	[59,60,67,68]
Honeydew and forest honeys	Frequently characterized by relatively high phenolic and mineral contents	High antioxidant capacity has been reported in several comparative studies	Composition varies according to plant and insect sources, geographical origin, and environmental conditions	[60,62,67]
Manuka honey (*Leptospermum scoparium*)	Characterized by methylglyoxal (MGO) together with phenolic constituents	Exhibits measurable antioxidant activity, although its distinctive biological profile is strongly associated with antibacterial activity	MGO content and antibacterial potency should not be interpreted as direct indicators of antioxidant or redox-modulating activity	[18]
Honey as a heterogeneous biological matrix	Phenolic acids and flavonoids are major contributors to antioxidant properties, together with other minor constituents	Antioxidant capacity generally correlates with phenolic composition, but results depend strongly on the assay used	Chemical antioxidant activity does not directly demonstrate bioavailability, cellular redox modulation, or neuroprotective efficacy	[57,63,64,67]

Abbreviations: MGO, methylglyoxal. Antioxidant capacities reported for different honey types should not be directly ranked across studies because of differences in sample origin, composition, preparation, and analytical methodology.

**Table 2:** Comparative characteristics of major bee-derived products relevant to redox modulation.

Bee-Derived Product	Representative Bioactive Constituents	Predominant Redox-Related Relevance	Evidence Base	Major Limitations	References
Honey	Phenolic acids, flavonoids, organic acids, enzymes, minerals, and other minor constituents	Chemical antioxidant activity is associated particularly with phenolic composition; antioxidant capacity varies markedly among botanical and geographical origins	Extensive chemical and in vitro antioxidant studies; comparative analyses of different honey types	High botanical and geographical variability; differences in processing and analytical methods; chemical antioxidant activity cannot be directly extrapolated to biological or neuroprotective effects	[53,57]
Propolis	Phenolic acids and esters, CAPE, flavonoids including pinocembrin, galangin, chrysin, apigenin, and quercetin	Strong chemical antioxidant activity together with experimental evidence of cellular redox and inflammatory modulation	Chemical analyses, in vitro and in vivo studies, and numerous reviews	Pronounced botanical and geographical heterogeneity; strong dependence on extraction method; insufficient standardization of preparations	[16,71]
Royal jelly	MRJPs, 10-HDA and other fatty acids, bioactive peptides, carbohydrates, vitamins, and minerals	Redox-related effects appear to involve modulation of cellular antioxidant and inflammatory responses rather than predominantly direct radical scavenging	Chemical, in vitro, and in vivo studies; limited human evidence	Composition affected by biological and environmental factors, processing, and storage; limited standardization and clinical evidence	[74,75,76,77]
Bee pollen and bee bread	Phenolic acids, flavonoids, carotenoids, phytosterols, unsaturated fatty acids, proteins, vitamins, and minerals	Antioxidant activity is frequently associated with phenolic and flavonoid content; transformation of pollen into bee bread may modify the accessibility of bioactive constituents	Chemical analyses, in vitro studies, comparative studies, and digestion models	Strong dependence on botanical origin; heterogeneous composition; variability associated with bee bread formation, extraction, and analytical methodology	[53,56]
Bee venom	Melittin, apamin, phospholipase A2, adolapin, mast cell-degranulating peptide, biogenic amines, and other peptides	Predominantly indirect redox modulation through effects on inflammatory, immune, and cellular stress responses; some preparations also show chemical radical-scavenging activity in vitro	In vitro and in vivo experimental studies; studies of whole venom and isolated components	Toxicity and allergenicity; marked dose dependence; differences between whole venom and isolated constituents; risk of severe hypersensitivity reactions	[78,80]
Beeswax	Long-chain esters, hydrocarbons, fatty acids, and trace amounts of phenolic compounds	Limited antioxidant relevance compared with other bee-derived products; minor chemical antioxidant activity has been reported	Primarily comparative chemical and analytical studies	Low abundance of redox-active compounds and very limited evidence of biologically relevant or neuroprotective effects	[53]

Abbreviations: CAPE, caffeic acid phenethyl ester; MRJPs, major royal jelly proteins; 10-HDA, 10-hydroxy-2-decenoic acid.

**Table 3:** Key determinants of the biological and translational relevance of bee-derived products and constituents.

Domain	Key Determinants or Processes	Relevance to Biological Interpretation	Main Limitations and Methodological Considerations	References
Chemical antioxidant activity	Radical-scavenging and reducing capacity measured using cell-free assays such as DPPH, ABTS, and FRAP	Useful for comparative chemical characterization, but does not directly demonstrate biological redox modulation or neuroprotective activity	Results depend strongly on assay conditions and product composition; high chemical antioxidant capacity should not be equated with in vivo efficacy	[53,81]
Bioaccessibility and gastrointestinal release	Release of bioactive constituents from the original matrix, solubility, digestion, and matrix-dependent accessibility for absorption	Determines the fraction of a compound potentially available for intestinal uptake after oral administration	Increased bioaccessibility does not necessarily indicate increased systemic bioavailability; effects are product- and compound-specific	[89,94]
Absorption, metabolism, and systemic exposure	Intestinal absorption, first-pass metabolism, conjugation, chemical stability, and clearance	Determines which parent compounds or metabolites reach the systemic circulation and at what concentrations	Circulating metabolites may differ substantially from compounds initially identified in the bee-derived product; data from isolated compounds cannot automatically be extrapolated to whole products	[82,92]
CNS and BBB relevance	Compound-specific physicochemical properties, systemic exposure, BBB transport, and potential interactions with cerebral vascular endothelium	Determines whether a constituent can directly reach the CNS or exert effects through vascular or peripheral mechanisms	BBB penetration cannot be inferred solely from lipophilicity, antioxidant activity, or the presence of a compound in a bee-derived product; direct pharmacokinetic or tissue-distribution evidence is required	[83,91]
Dose, formulation, and route of administration	Dose, chemical form, formulation, stability, and oral versus parenteral administration	Strongly influences exposure and may alter the relationship between experimental dose and biologically achievable concentrations	Findings obtained with purified compounds, specialized delivery systems, or injected bee venom may not reflect exposure following consumption of the corresponding natural product	[87,90]
Product characterization and standardization	Botanical and geographical origin, processing, extraction procedure, chemical fingerprinting, marker compounds, and batch-to-batch consistency	Essential for reproducibility and for attributing biological effects to a defined preparation	High compositional heterogeneity and differences in extraction and analytical methodology limit direct comparison among studies; marker compounds should be appropriate for the specific product or chemotype	[54,93]

Abbreviations: ABTS, 2,2′-azino-bis(3-ethylbenzothiazoline-6-sulfonic acid); BBB, blood–brain barrier; CNS, central nervous system; DPPH, 2,2-diphenyl-1-picrylhydrazyl; FRAP, ferric reducing antioxidant power.

**Table 4:** Representative mechanistic evidence for neuroprotective and redox-modulating effects of bee-derived products, associated constituents, and related experimental preparations.

Mechanistic Domain	Investigated Agent	Bee-Product Association/Type of Preparation	Molecular Target or Process	Experimental Model	Main Mechanistic Finding	References
Nrf2/ARE antioxidant response	Pinocembrin	Honey- and propolis-associated flavonoid; isolated compound	Nrf2/ARE	6-OHDA-treated SH-SY5Y cells	Activation of Nrf2/ARE signaling and protection against oxidative neuronal injury	[97]
Nrf2/ARE antioxidant response	Pinocembrin	Honey- and propolis-associated flavonoid; isolated compound	ERK1/2/HO-1	MPP+-treated SH-SY5Y cells	Induction of HO-1 through ERK1/2 signaling and attenuation of neurotoxicity	[98]
Nrf2/ARE antioxidant response	Melittin	Major bee venom peptide; isolated constituent	Keap1/Nrf2/HO-1	Glutamate-stressed HT22 hippocampal cells	Activation of antioxidant signaling associated with reduced oxidative and inflammatory injury	[99]
Nrf2/ARE antioxidant response	HPO-DAEE	Synthetic derivative related to royal jelly fatty-acid chemistry	Nrf2/ARE and eIF2α/ATF4	6-OHDA-treated SH-SY5Y cells	Induction of HO-1 and protection against 6-OHDA-induced cell death	[100]
Nrf2/ARE antioxidant response	FA-97	Synthetic CAPE derivative	Nrf2/HO-1	Neuronal oxidative-stress model and scopolamine-induced cognitive impairment model	Reduction in oxidative stress-mediated neuronal apoptosis and improvement of cognitive impairment	[101]
Nrf2/ARE antioxidant response	CAPE	Propolis-associated phenolic ester; isolated compound	Nrf2/HO-1	Aβ1–42 oligomer-injected mice	Nrf2/HO-1 activation associated with neuroprotection and reduced glial activation	[102]
NF-κB-dependent inflammatory signaling	Bee venom	Whole bee venom preparation	NF-κB signaling	LPS-induced neuroinflammation and memory-impairment model	Suppression of NF-κB activation and attenuation of inflammatory responses	[104]
NF-κB-dependent inflammatory signaling	Bee venom	Whole bee venom preparation	MyD88-dependent NF-κB signaling	LPS-activated BV2 microglial cells	Reduced NF-κB nuclear translocation and decreased expression of COX-2, TNF-α, IL-1β, and IL-6	[105]
Calcium-dependent and inflammatory signaling	Apamin	Bee venom peptide; isolated constituent	CaMKII/ERK/p65/STAT3 signaling	MPP+-treated SH-SY5Y cells and primary mesencephalic neurons	Attenuation of calcium-overload-related signaling and dopaminergic neuronal injury	[106]
NLRP3 inflammasome modulation	CAPE	Propolis-associated phenolic ester; isolated compound	ROS generation and NLRP3 inflammasome-related signaling	Zinc-deficiency-stressed SIM-A9 microglial and SH-SY5Y neuronal cells	Attenuation of ROS production and NLRP3 inflammasome-related responses	[107]
NLRP3 inflammasome and mitochondrial quality control	Apigenin	Propolis-associated flavonoid; isolated compound	Mitophagy, ROS/NLRP3 signaling, and pyroptosis	Experimental spinal cord injury in rats	Promotion of mitophagy, reduction in ROS accumulation, and suppression of NLRP3 activation and pyroptosis	[109]
Mitochondrial protection	Algerian propolis extract	Whole-product extract	Mitochondrial membrane potential	Ferulenol-treated isolated rat liver mitochondria	Restoration of ferulenol-induced loss of mitochondrial membrane potential	[111]
Mitochondrial protection	CAPE	Propolis-associated phenolic ester; isolated compound	UCP2-dependent mitochondrial redox regulation	Oxidative-stress-exposed ARPE-19 retinal pigment epithelial cells	UCP2-dependent attenuation of basal and stress-induced ROS generation	[112]
Anti-apoptotic and pro-survival signaling	Pinocembrin	Honey- and propolis-associated flavonoid; isolated compound	Bax/Bcl-2 balance	Glutamate-treated SH-SY5Y cells	Decreased Bax/Bcl-2 ratio, reduced LDH release, and attenuation of apoptosis	[113]
Anti-apoptotic and pro-survival signaling	Bee venom	Whole bee venom preparation	PI3K/Akt	MPP+-treated SH-SY5Y cells	Reduced apoptotic cell death and modulation of Bcl-2/Bax signaling, with PI3K/Akt involvement	[114]
Anti-apoptotic and pro-survival signaling	CAPE	Propolis-associated phenolic ester; isolated compound	p38 MAPK/Nrf2/HO-1 and BDNF	Rat organotypic midbrain slice cultures	Protection of dopaminergic neurons associated with p38 MAPK-dependent Nrf2 activation and induction of HO-1 and BDNF	[115]
Anti-apoptotic and pro-survival signaling	CAPE	Propolis-associated phenolic ester; isolated compound	MAPK and Akt/GSK3β signaling	Acrolein-treated HT22 hippocampal cells	Attenuation of ROS accumulation and GSH depletion and reduction in neurotoxicity	[116]
Proteostasis and protein aggregation	Bee venom	Whole bee venom preparation	Ubiquitin–proteasome system	hSOD1G85R-expressing NSC34 motor neuron-like cells	Restoration of proteasome activity and reduced accumulation of ubiquitinated mutant SOD1 and misfolded SOD1	[118]
Proteostasis and protein aggregation	Propolis polyphenol-based nanosheets	Engineered material derived from propolis polyphenols	α-Synuclein fibril assembly	Experimental α-synuclein aggregation model	Inhibition of α-synuclein amyloid aggregation and redirection toward less cytotoxic off-pathway oligomeric species	[119]
Amyloidogenic processing	Purified royal jelly-derived peptide fraction	Peptide fraction derived from royal jelly	BACE1-related amyloidogenic processing	N2a/APP695swe cells	Downregulation of BACE1 and reduction in extracellular Aβ1–40 and Aβ1–42 production	[120]
Amyloidogenic processing—computational evidence	Rutin, 3,4-dicaffeoylquinic acid, nemorosone, luteolin, and other candidate phytochemicals	Bee-product-associated phytochemicals evaluated computationally	Predicted BACE1 binding	In silico molecular screening	Identification of candidate high-affinity BACE1 ligands requiring experimental validation	[121]

Abbreviations: 6-OHDA, 6-hydroxydopamine; Aβ, amyloid-β; APP, amyloid precursor protein; ARE, antioxidant response element; ASC, apoptosis-associated speck-like protein containing a CARD; ATF4, activating transcription factor 4; BACE1, β-site amyloid precursor protein-cleaving enzyme 1; Bax, Bcl-2-associated X protein; Bcl-2, B-cell lymphoma 2; BDNF, brain-derived neurotrophic factor; CAPE, caffeic acid phenethyl ester; CaMKII, Ca^2+^/calmodulin-dependent protein kinase II; COX-2, cyclooxygenase-2; eIF2α, eukaryotic translation initiation factor 2α; ERK, extracellular signal-regulated kinase; GSH, glutathione; GSK3β, glycogen synthase kinase 3β; HO-1, heme oxygenase-1; HPO-DAEE, 4-hydroperoxy-2-decenoic acid ethyl ester; IL, interleukin; Keap1, Kelch-like ECH-associated protein 1; LDH, lactate dehydrogenase; LPS, lipopolysaccharide; MAPK, mitogen-activated protein kinase; MPP+, 1-methyl-4-phenylpyridinium; MyD88, myeloid differentiation primary response 88; NF-κB, nuclear factor κB; NLRP3, NLR family pyrin domain containing 3; Nrf2, nuclear factor erythroid 2-related factor 2; PI3K, phosphoinositide 3-kinase; ROS, reactive oxygen species; SOD1, superoxide dismutase 1; STAT3, signal transducer and activator of transcription 3; TNF-α, tumor necrosis factor-α; UCP2, uncoupling protein 2. Evidence summarized in the table includes whole bee-derived products, isolated constituents, synthetic derivatives, engineered preparations, and computational studies; these categories should not be interpreted as providing equivalent levels of evidence.

**Table 5:** Preclinical evidence for bee-derived products and constituents in Alzheimer’s disease models.

Product or Constituent	Experimental Model/Intervention	Main Findings	Associated Mechanism or Biological Changes	Evidence Type	References
CAPE, i.p.	Aβ1–42 oligomer-injected mice	Attenuation of memory impairment and hippocampal neuronal loss	Nrf2/HO-1 activation, modulation of GSK3β, reduced oxidative stress, apoptosis, and glial activation	Animal model	[102]
CAPE, i.p.	STZ-ICV Wistar rat model	Improved cognitive performance; prevention of the STZ-induced increase in TBARS; increased GSH	Attenuation of oxidative stress and TNF-α-associated inflammatory responses	Animal model	[123]
CAPE, i.p.	STZ-ICV Wistar rat model	Attenuation of memory impairment	PI3K-dependent nitric oxide signaling; modulation of oxidative stress, eNOS, TNF-α, NF-κB, and AChE	Animal model	[124]
CAPE	Tau-expressing *Drosophila melanogaster* and scopolamine-induced mouse model of cognitive impairment	Improvement of selected disease-related behavioral and physiological outcomes	Neurodifferentiation-associated effects; increased BDNF, Nestin, and NeuN in the mouse model	Invertebrate and animal models	[125]
bvPLA2	Tg2576 mice	Improved memory and reduced Aβ-related and neuroinflammatory alterations	Reduced APP, BACE1, and Aβ; inhibition of STAT3 signaling and inflammatory responses	Animal model	[126]
bvPLA2	3xTg-AD mice	Improved cognitive function, reduced hippocampal Aβ deposition, and increased brain glucose metabolism	Increased regulatory T-cell responses, microglial deactivation, and reduced CD4+ T-cell infiltration	Animal model	[127]
bvPLA2	Aβ-vaccinated 3xTg-AD mice	Reduced Aβ burden and cognitive deficits without exacerbation of vaccination-associated CNS inflammation	Modulation of regulatory T-cell responses and attenuation of inflammatory cell infiltration	Animal model	[128]
bvPLA2	LPS-induced AD-like mouse model	Improved memory and reduced Aβ-related and inflammatory alterations	Reduced amyloidogenic proteins and inflammatory mediators through inhibition of NF-κB signaling	Animal model	[129]
Whole bee venom	LPS-induced mouse model of memory impairment and amyloidogenesis	Reduced memory impairment, Aβ accumulation, and neuroinflammatory alterations	Inhibition of NF-κB signaling, secretase activity, and expression of inflammatory proteins	Animal model	[104]
Royal jelly	ICV-STZ rat model; royal jelly incorporated into the diet	Improvement of spatial learning and memory	Mechanism not directly established in the study	Animal model	[130]
Royal jelly, oral	ICV-STZ Wistar rat model	Improved working memory and reduced hippocampal neurodegeneration	Reduced oxidative stress and increased hippocampal cell proliferation/neurogenesis-related changes	Animal model	[131]
Indian royal jelly, oral	Okadaic acid-induced rat model	Improved spatial and recognition memory and attenuation of neuroinflammatory and oxidative alterations	Reduced oxidative stress and inflammatory cytokines; increased cholinergic tone; in silico interaction of 10-HDA with tau-related kinases and phosphatases	Animal model with complementary in silico analysis	[132]
Royal jelly	APP/PS1 mice	Improved cognitive performance, reduced Aβ accumulation, and reduced neuronal apoptosis	Activation of cAMP/PKA/CREB/BDNF signaling and attenuation of oxidative stress/JNK-associated apoptosis	Animal model	[133]
Royal jelly	Rabbit model induced by 2% cholesterol diet and copper-containing drinking water	Reduced cerebral Aβ pathology and enhanced neuronal metabolic activity	Reduced BACE1 and RAGE; increased LRP-1 and IDE; enhanced antioxidant capacity and reduced caspase-3 activation	Animal model	[134]
Royal jelly, oral gavage	ICV Aβ1–40-induced rat model	Improved learning and memory	Attenuation of hippocampal oxidative stress, including reduced MDA and TOS and increased TAC	Animal model	[135]
Royal jelly and enzyme-treated royal jelly	Aβ-expressing *C. elegans* CL2006 model	Delayed Aβ-associated paralysis and improved proteostasis	DAF-16-dependent modulation of proteostasis within insulin/IGF signaling	Invertebrate model	[136]

Abbreviations: Aβ, amyloid-β; AChE, acetylcholinesterase; APP, amyloid precursor protein; BACE1, β-site amyloid precursor protein-cleaving enzyme 1; BDNF, brain-derived neurotrophic factor; bvPLA2, bee venom phospholipase A2; CAPE, caffeic acid phenethyl ester; CNS, central nervous system; eNOS, endothelial nitric oxide synthase; GSH, glutathione; GSK3β, glycogen synthase kinase 3β; HO-1, heme oxygenase-1; ICV, intracerebroventricular; IDE, insulin-degrading enzyme; LPS, lipopolysaccharide; LRP-1, low-density lipoprotein receptor-related protein 1; MDA, malondialdehyde; NF-κB, nuclear factor κB; Nrf2, nuclear factor erythroid 2-related factor 2; RAGE, receptor for advanced glycation end products; ROS, reactive oxygen species; STAT3, signal transducer and activator of transcription 3; STZ, streptozotocin; TAC, total antioxidant capacity; TBARS, thiobarbituric acid-reactive substances; TNF-α, tumor necrosis factor-α; TOS, total oxidant status. All studies summarized in this table are preclinical; evidence obtained from transgenic, chemically induced, and invertebrate models should not be interpreted as demonstrating therapeutic efficacy in human Alzheimer’s disease.

**Table 6:** Preclinical and clinical evidence for bee-derived products, constituents, and bee venom-based interventions in Parkinson’s disease.

Product or Constituent	Experimental Model/Intervention	Main Findings	Associated Mechanism or Biological Changes	Evidence Type	Reference
CAPE	MPTP mouse model	Attenuation of dopaminergic neurodegeneration and striatal dopamine depletion	Reduced iNOS and cell-death-related signaling	Animal model	[139]
CAPE	6-OHDA rat model	Protection against dopaminergic neuronal loss	Inhibition of mitochondrial permeability transition and attenuation of oxidative injury	Animal model	[84]
CAPE	6-OHDA-treated rat rostral mesencephalic and cerebellar granule neurons	Reduced free-radical generation and neuronal death	Attenuation of 6-OHDA-induced oxidative injury	Cellular model	[140]
CAPE	Experimental dopaminergic injury models involving organotypic midbrain cultures and in vivo neurotoxic injury	Preservation of dopaminergic neurons and improvement of behavioral outcomes	p38 MAPK-dependent Nrf2 activation; induction of HO-1 and BDNF; reduced microglial/macrophage activation	Ex vivo and animal models	[115]
Propolis ± levodopa	*PINK1B9 Drosophila melanogaster* parkinsonism model	Improved motor-related outcomes and lifespan	Increased CAT activity and reduced ROS accumulation	Invertebrate model	[141]
Chrysin	6-OHDA-treated mice	Attenuation of behavioral abnormalities and neuroinflammatory alterations	Improved antioxidant-related parameters and reduced inflammatory signaling	Animal model	[142]
Liquiritigenin-rich Brazilian red propolis extract	6-OHDA hemiparkinsonian rat model with L-DOPA-induced dyskinesia	Reduction in L-DOPA-induced dyskinesia	Astrocyte-associated changes and preservation of dopaminergic-related markers	Animal model	[143]
Propolis polyphenol-based nanosheets	Rotenone-induced cellular and rat PD models	Neuroprotection and improvement of neurobehavioral and neurochemical outcomes	Reduced lipid peroxidation; increased GSH and antioxidant enzyme activity	Cellular and animal models; engineered formulation	[144]
10-HDA (queen bee acid)	Neuronal cellular systems, PD-related mouse model, and *D. melanogaster*	Autophagy-associated neuroprotection and increased lifespan in *Drosophila*	Beclin-1/mTOR- and SIRT1-associated induction of autophagy	Cellular, animal, and invertebrate models	[145]
Bee venom acupuncture	Acute MPTP mouse model	Preservation of nigral dopaminergic neurons and striatal dopaminergic fibers	Attenuation of stress-related signaling, including c-Jun activation	Animal model	[156]
Whole bee venom	MPTP mouse model	Reduced dopaminergic neuronal loss	Reduced MAC-1 and iNOS expression and attenuation of microglial activation	Animal model	[157]
Whole bee venom	MPTP mouse model	Prevention of dopaminergic neuronal degeneration	Microglial deactivation, reduced CD4+ T-cell infiltration, and induction of CD4+CD25+Foxp3+ regulatory T cells	Animal model	[148]
Apamin/whole bee venom	Chronic MPTP/probenecid mouse model	Apamin exerted neuroprotection, but protection was weaker than that produced by whole bee venom	Partial reproduction of whole-venom neuroprotection, suggesting contributions from additional venom constituents	Animal model	[149]
Bee venom acupuncture	Rotenone-treated mice	Improved locomotor performance and restoration of brain neurochemical alterations	Reduced oxidative stress, neuroinflammation, DNA damage, and apoptosis	Animal model	[146]
Whole bee venom	Rotenone-treated mice	Improved motor performance and attenuated neurodegenerative alterations	Reduced oxidative/nitrosative stress, including lower MDA and NO and preservation of GSH	Animal model	[147]
bvPLA2	A53T α-synuclein transgenic mice	Improved motor dysfunction and reduced α-synuclein-associated pathology	Reduced microglial activation and modulation of the M1/M2 microglial balance	Animal model	[150]
Standardized bvPLA2	MPTP-treated mice	Dose-dependent improvement of motor deficits and preservation of dopaminergic neurons	Induction of CD4+CD25+Foxp3+ regulatory T cells, suppression of Th1/Th17 responses, and reduced microglial activation	Animal model; standardized GMP-produced preparation	[151]
Whole bee venom	Reserpine-induced PD rat model; bee venom compared separately with L-DOPA and dopamine-loaded nanoparticles	Restoration of monoaminergic and neurotransmitter alterations and attenuation of cellular injury	Reduced DNA fragmentation, IL-1β and TNF-α; increased PON1 and BDNF	Animal model	[158]
Whole bee venom, s.c.	Randomized double-blind placebo-controlled trial; monthly injections for 11 months	No significant symptomatic or disease-modifying benefit compared with placebo	No significant between-group improvement in motor outcomes or imaging evidence of disease progression	Human clinical RCT	[152]
Acupuncture + bee venom acupuncture	Prospective open-label adjunctive study in patients with idiopathic PD	Improvements in gait speed, activities of daily living, quality of life, and motor symptoms	Mechanism not directly established; multimodal intervention	Human clinical, open-label	[153]
Acupuncture + bee venom acupuncture	Single-center, double-blind, three-arm randomized controlled trial	Improvement in selected motor, activities-of-daily-living, and gait-related outcomes	Multimodal intervention; bee venom-specific contribution cannot be isolated	Human clinical RCT	[154]
Acupuncture + bee venom acupuncture	Double-blind randomized neuroimaging study in patients with idiopathic PD	Changes in regional cerebral perfusion; increase in striatal dopamine transporter binding was not significantly different between groups	Exploratory changes in dopamine availability and cerebral blood flow	Human clinical, randomized mechanistic study	[155]

Abbreviations: 6-OHDA, 6-hydroxydopamine; BDNF, brain-derived neurotrophic factor; bvPLA2, bee venom phospholipase A2; BVA, bee venom acupuncture; CAPE, caffeic acid phenethyl ester; CAT, catalase; DA, dopamine; GFAP, glial fibrillary acidic protein; GSH, glutathione; GMP, good manufacturing practice; HO-1, heme oxygenase-1; iNOS, inducible nitric oxide synthase; IL, interleukin; L-DOPA, levodopa; LPS, lipopolysaccharide; MDA, malondialdehyde; MPTP, 1-methyl-4-phenyl-1,2,3,6-tetrahydropyridine; NF-κB, nuclear factor κB; NO, nitric oxide; Nrf2, nuclear factor erythroid 2-related factor 2; PD, Parkinson’s disease; PON1, paraoxonase 1; ROS, reactive oxygen species; SIRT1, sirtuin 1; TH, tyrosine hydroxylase; TNF-α, tumor necrosis factor-α; Treg, regulatory T cell. Preclinical studies summarized in this table involve heterogeneous whole products, isolated constituents, routes of administration, and experimental formulations and should not be regarded as equivalent interventions. Clinical studies of acupuncture combined with bee venom acupuncture represent multimodal interventions and cannot be directly compared with trials of subcutaneous bee venom alone.

**Table 7:** Preclinical evidence for bee-derived products and constituents in amyotrophic lateral sclerosis and related experimental models.

Product or Constituent	Experimental Model/Intervention	Main Findings	Associated Mechanism or Biological Changes	Evidence Type	References
Bee venom	Symptomatic hSOD1G93A mice; subcutaneous BV administration at bilateral ST36 acupoints	Improved motor performance and prolonged survival	Reduced microglial activation and phospho-p38 MAPK; decreased caspase-3 activity; preservation of mitochondrial ultrastructure	Animal model	[160]
Bee venom acupuncture	Symptomatic hSOD1G93A mice; BVA at ST36 compared with intraperitoneal BV administration	Improved motor function and preservation of spinal motor neurons	Reduced spinal TLR4-, CD14-, and TNF-α-associated inflammatory signaling; central effects differed according to route/site of administration	Animal model	[161]
CAPE	Symptomatic hSOD1G93A mice; oral administration initiated after disease onset	Prolonged post-onset survival and overall lifespan; increased motor neuron preservation	Reduced microglial and astrocytic activation and decreased phospho-p38 MAPK in the spinal cord	Animal model	[162]
Brazilian green propolis extract; kaempferol and kaempferide	Mutant SOD1G85R-expressing N2a cells	Reduced mutant SOD1-associated neurotoxicity and intracellular SOD1 aggregation	Autophagy-associated clearance of mutant SOD1; kaempferol and kaempferide reduced mitochondrial superoxide, while kaempferol additionally activated AMPK/mTOR-dependent autophagy	Cellular model	[163]
p-Coumaric acid	Mutant SOD1-expressing N2a cells	Reduced mutant SOD1-associated cellular toxicity	Activation of autophagy and enhanced clearance of mutant SOD1-associated protein stress	Cellular model	[164]
Coffee honey from *Apis cerana*	Ubiquilin-knockdown *Drosophila melanogaster* model	Improved locomotion and learning; partial rescue of neuromuscular-junction abnormalities; reduced ROS accumulation in larval brains	Reduction in oxidative stress and partial improvement of neuronal functional abnormalities	Invertebrate ALS/FTD-related model	[165]
Melittin from *Apis dorsata*	Ubiquilin-knockdown *Drosophila melanogaster* model	Improved locomotor function, but no significant improvement in learning or the examined neuromuscular-junction abnormalities	Mechanism responsible for locomotor improvement not established	Invertebrate ALS/FTD-related model	[165]

Abbreviations: ALS, amyotrophic lateral sclerosis; AMPK, AMP-activated protein kinase; BVA, bee venom acupuncture; CAPE, caffeic acid phenethyl ester; FTD, frontotemporal dementia; MAPK, mitogen-activated protein kinase; mTOR, mechanistic target of rapamycin; ROS, reactive oxygen species; SOD1, superoxide dismutase 1; TLR4, Toll-like receptor 4; TNF-α, tumor necrosis factor-α. All evidence summarized in this table is preclinical and is derived predominantly from mutant SOD1-based models or an ALS/FTD-related invertebrate model. These experimental systems reproduce selected aspects of ALS pathobiology and should not be interpreted as evidence of therapeutic efficacy in the heterogeneous human disease.

**Table 8:** Evidence for bee-derived products and constituents in complementary neurological models relevant to neurodegeneration.

Product or Constituent	Experimental Model/Intervention	Main Findings	Associated Mechanism or Biological Changes	Evidence Type	References
Malaysian Tualang honey	Chronic cerebral hypoperfusion induced by permanent bilateral common carotid artery occlusion in rats	Reduced hippocampal CA1 neuronal loss and preservation of tissue morphology	Molecular mechanism was not directly investigated in the study	Animal model	[166]
Brazilian green propolis	Permanent middle cerebral artery occlusion in mice	Reduced cerebral infarct volume and brain swelling	Antioxidant activity was demonstrated in complementary experimental assays; its contribution to the in vivo anti-ischemic effect remains inferential	Animal model	[167]
Royal jelly hydrolysate (hydrolase-treated royal jelly)	H_2_O_2_- and glutamate-treated SH-SY5Y cells and a rat model of vascular dementia induced by right middle cerebral artery occlusion	Protection against oxidative/excitotoxic cell injury and improvement of spatial memory	Reduced hippocampal acetylcholinesterase activity together with direct cellular cytoprotection	Cellular and animal models	[168]
CAPE	Aged Sprague–Dawley rats receiving chronic CAPE administration	Reduced MDA levels and attenuation of age-associated ultrastructural alterations in the brain and cerebellum	Improvement of selected endogenous antioxidant defense parameters and attenuation of age-related oxidative damage	Animal model	[169]
CAPE	H_2_O_2_-stressed 661W photoreceptor-derived cells and acute retinal light-stress model in rats	Reduced oxidative cell injury and improved retinal functional responses	Increased HO-1 expression, reduced NF-κB nuclear translocation, and modulation of oxidative/inflammatory signaling	Cellular and animal retinal models	[170]

Abbreviations: AChE, acetylcholinesterase; CAPE, caffeic acid phenethyl ester; HO-1, heme oxygenase-1; MDA, malondialdehyde; NF-κB, nuclear factor κB; ROS, reactive oxygen species. The models summarized in this table represent vascular, ischemic, aging-related, and retinal experimental conditions and should be regarded as complementary evidence for neuroprotective or redox-modulating activity rather than as direct models of chronic neurodegenerative diseases such as Alzheimer’s disease, Parkinson’s disease, or amyotrophic lateral sclerosis.

## Data Availability

No new data were created or analyzed in this study. Data sharing is not applicable to this article.
